# Fast Pairwise Structural RNA Alignments by Pruning of the Dynamical Programming Matrix

**DOI:** 10.1371/journal.pcbi.0030193

**Published:** 2007-10-12

**Authors:** Jakob H Havgaard, Elfar Torarinsson, Jan Gorodkin

**Affiliations:** 1 Division of Genetics and Bioinformatics, University of Copenhagen, Frederiksberg, Denmark; 2 Department of Natural Sciences, University of Copenhagen, Frederiksberg, Denmark; Center for Human Genetics and Molecular Pediatric Disease, United States of America

## Abstract

It has become clear that noncoding RNAs (ncRNA) play important roles in cells, and emerging studies indicate that there might be a large number of unknown ncRNAs in mammalian genomes. There exist computational methods that can be used to search for ncRNAs by comparing sequences from different genomes. One main problem with these methods is their computational complexity, and heuristics are therefore employed. Two heuristics are currently very popular: pre-folding and pre-aligning. However, these heuristics are not ideal, as pre-aligning is dependent on sequence similarity that may not be present and pre-folding ignores the comparative information. Here, pruning of the dynamical programming matrix is presented as an alternative novel heuristic constraint. All subalignments that do not exceed a length-dependent minimum score are discarded as the matrix is filled out, thus giving the advantage of providing the constraints dynamically. This has been included in a new implementation of the FOLDALIGN algorithm for pairwise local or global structural alignment of RNA sequences. It is shown that time and memory requirements are dramatically lowered while overall performance is maintained. Furthermore, a new divide and conquer method is introduced to limit the memory requirement during global alignment and backtrack of local alignment. All branch points in the computed RNA structure are found and used to divide the structure into smaller unbranched segments. Each segment is then realigned and backtracked in a normal fashion. Finally, the FOLDALIGN algorithm has also been updated with a better memory implementation and an improved energy model. With these improvements in the algorithm, the FOLDALIGN software package provides the molecular biologist with an efficient and user-friendly tool for searching for new ncRNAs. The software package is available for download at http://foldalign.ku.dk.

## Author Summary

FOLDALIGN is an algorithm for making pairwise structural alignments of RNA sequences. It uses a lightweight energy model and sequence similarity to simultaneously fold and align the sequences. The algorithm can make local and global alignments. The power of structural alignment methods is that they can align sequences where the primary sequences have diverged too much for normal alignment methods to be useful. The structures predicted by structural alignment methods are usually better than the structures predicted by single-sequence folding methods since they can take comparative information into account. The main problem for most structural alignment methods is that they are too computationally expensive. In this paper we introduce the dynamical pruning heuristic that makes the FOLDALIGN method significantly faster without lowering the predictive performance. The memory requirements are also significantly lowered, allowing for the analysis of longer sequences. A user-friendly (still command-line based, though) implementation of the algorithm is available at the Web site: http://foldalign.ku.dk

## Introduction

Noncoding RNA (ncRNA) genes and regulatory structures have been shown to be both highly abundant and highly diverse parts of the genome [1,2]. One theory is that many of these ncRNAs are part of RNA-based regulatory systems [3]. Recently, several papers about large-scale searches for vertebrate RNA genes or motifs using comparative genomics have been published [4–6]. These large-scale searches indicate that there are potentially many unknown structures still hidden in the genomes.

It has been shown that alignment of ncRNAs requires information about secondary structure when the sequence similarity is below 60% [7]. The reason for this is that compensating mutations change the primary sequence without changing the structure of the molecule. The Sankoff algorithm for simultaneously folding and aligning of RNA sequences can in principle be applied to cope with this [8]. However, the resource requirements of the algorithm are too high even for a few short sequences. For two sequences of length *L*, the time complexity is *O*(*L* ^6^) and the memory complexity is *O*(*L* ^4^). Heuristics are therefore needed before the algorithms for folding and aligning RNA sequences become fast enough to be useful.

FOLDALIGN 1.0 was the first simplified implementation of the Sankoff algorithm [9]. It contained a simple scoring scheme with separate substitution matrices for base-paired and single-stranded nucleotides. It had three constraints: (i) the length of the final alignment could not be longer than λ nucleotides; (ii) the maximum length difference between two subsequences being aligned was limited to δ nucleotides, and (iii) it could only align stem-loop structures. The second version of the algorithm uses a combination of substitutions (similar to the RIBOSUM matrices [10]) and a lightweight energy model to align two sequences [11]. FOLDALIGN 2.0 also uses the λ and δ constraints, but it can align branched structures. This algorithm was used in one of the large-scale searches for vertebrate ncRNAs [6].

Variants of two types of heuristics are currently very popular, namely pre-aligning and pre-folding. The pre-aligning methods use sequence similarity to limit the search space by requiring that the final alignment must contain the pre-aligned nucleotides. The length of the pre-aligned subsequences varies from short stretches called anchors [12] to full sequences [13–16]. Methods that require the sequences to be fully aligned before the structure is predicted are not strictly Sankoff-based methods, as they separate the alignment and folding steps completely. Pre-folding uses single-sequence folding to limit the structures that can be found by the comparative algorithms. A popular method of pre-folding is to use base-pairing probabilities found by the single-sequence folding to limit which base pairs can be included in the conserved structure [17,18]. As for align-then-fold methods, methods using pre-folding can be taken to the extreme where the folding and alignment steps are completely separated. One example of this is the combination of the RNAcast and the RNAforester methods [19,20]. Some methods can use both pre-aligning and pre-folding heuristics [21–23].

The currently implemented Sankoff-based methods, for pairwise alignment and secondary-structure prediction of RNA sequences, can be split into two groups: the energy-based methods and the probabilistic methods. The energy-based methods, FOLDALIGN, Dynalign [24], locaRNA [17], and SCARNA [23], are based on minimization of the free energy [25]. Free-energy minimization is based on a physical model of how the different elements of an RNA structure contribute to the free energy. The parameters are partly found experimentally and partly estimated from multiple alignments. The probabilistic models are usually based on Stochastic Context Free Grammars (SCFGs); see [26] for an introduction. These methods include Consan [12] and Stemloc [22]. The Stochastic Context Free Grammars parameters are estimated from multiple alignments. Each of these methods uses different kinds of heuristics. The previous version of FOLDALIGN [11] uses banding (δ, see below), limits the alignment length for local alignments (λ, see below), and limits the number of ways a bifurcation can be calculated (described below). Dynalign [24] uses banding based on pre-alignment using a hidden Markov model. LocaRNA [17] limits the number of potential base pairs by only using base pairs with a single-sequence base-pair probability above a given cutoff. SCARNA [23] uses a similar strategy, but further decouples the left and right sides of the base pairs. Consan [12] uses short stretches of normal sequence alignments to constrain the folding. Stemloc [22] uses the *N*_1_ best single-sequence–predicted structures and the *N*_2_ best normal-sequence alignments to limit the final combined alignment and structure prediction.

In this paper, dynamical pruning of the dynamic programming matrix is introduced as a new heuristic in the FOLDALIGN algorithm [11]. In all its simplicity, the dynamic pruning discards any subalignment that does not have a score above a length-dependent threshold. This is similar to one of the heuristics used in BLAST [27]. The advantage of the pruning method compared with the pre-aligning methods is that it can be used when there is not enough sequence similarity to make the necessary alignments. The advantage compared with the pre-folding methods is that none of the comparative information is lost in a single-sequence folding step. It is shown empirically that the pruning leads to a huge speed increase while the algorithm retains its good performance. The speed increase makes studies like [6] much more feasible. The method of dynamical pruning is simple and general. It should therefore be possible to use it in many of the other methods available for folding and aligning RNA sequences. As pruning is a feature of the dynamic programming method, it may be used in any algorithm using dynamic programming.

In addition to the dynamical pruning, the FOLDALIGN software package has been significantly updated. The constraint, which speeds up the algorithm by limiting the calculation of branch points, is now also used to lower the memory requirement during the local-alignment stage. During the backtrack stage of the algorithm, more information is needed. To try to limit the memory consumption during this stage, an extra pre-backtrack step is used. The pre-backtrack step locates all branch points in the conserved structure, and these are then used to divide the structure into unbranched segments. The unbranched structures are then realigned and backtracked separately. As the unbranched structures usually are shorter than the full branched structure, the memory consumption is reduced. The use of the divide and conquer method increases the run time of the algorithm, but not by much since the realignments of the segments are unbranched.

In addition to the algorithmic improvements, the energy model has been improved as well. External single-strand nucleotides are scored in a consistent way. Also, more insert base pairs are allowed. These improvements lead to better structure predictions.

## Results

FOLDALIGN is a tool for making local or global structural alignments of RNAs [9,11,28–31]. It uses a combination of sequence similarity and structure to make the alignment. The present article shows how the two main inconveniences, the time and memory requirements, can be drastically lowered without losing the ability to make good alignments.

The main improvement of the algorithm is the dynamical pruning. The pruning eliminates subalignments that are so poor that they can be assumed to never be a part of a biologically relevant alignment [27]. The pruning works by eliminating all subalignments with a score below a length-dependent threshold. This not only removes the subalignment itself but also all the longer alignments that the subalignment would have become a part of. Elimination of subalignments also improves the memory performance since it is not necessary to store the eliminated alignments. The dynamical pruning method is general to the dynamic programming method, and it should therefore be trivial to use it in other applications where dynamic programming is used. Note however, that when pruning is used there is no guarantee that the solution is the optimal solution, and in some cases a solution is not found at all. In these rare cases pruning is not feasible, but FOLDALIGN can provide an alignment by realigning without pruning.

The memory requirement of the algorithm is further lowered by exploiting the branch point constraint. In [11] the calculation of branch points was limited to one calculation for equivalent alignments/structures. This speeds up the algorithm significantly. Here, it is also used to lower the memory requirement; see below and the Memory implementation section of Protocol S1.

Much effort has been put into trying, during backtrack, to keep the memory requirement below what is needed during the initial local-alignment scan. This is done by using a divide and conquer scheme that first locates all branch points in the structure, and then uses them to divide the structure into hopefully shorter unbranched segments. These segments are then backtracked normally. While this strategy usually works, there is no guarantee that it will always keep the memory usage low during backtrack. One clear example where it does not work is in the case of an unbranched alignment. A cubic space model similar to the linear space models [32,33] used in sequence alignment could be used to keep the memory requirement below a given cutoff. A method similar to the Treeterbi method [34], of locating and removing memory cells that are not part of final alignment during the alignment step, could also be used to limit the memory consumption during the realignment of stem segments.

### Algorithms and Heuristics

The algorithm uses a lightweight energy model based on energy minimization [11,25] and sequence similarity to simultaneously fold and align two sequences. The energy model has five different contexts: stem, hairpin-loop, bulge-loop, internal-loop, and external/bifurcated-loop. In the stem context, two pairs of nucleotides are allowed to base-pair if both pairs can form an *A* ⇔ *U*, *C* ⇔ *G*, or *G* ⇔ *U* base pair. A stem must always start with such a conserved base pair, but if the stem is already at least one base-pair long, then a base pair in one of the sequences can be aligned to two gaps in the other sequence. A stem must contain at least two conserved base pairs. The four other contexts are used to align unpaired nucleotides; see Protocol S1 and [11] for details. The parameters of the energy model have all been multiplied by −10 mol/kcal to allow the maximization of the alignment score [11]. Different sequence similarity substitution scores are used for base-paired nucleotides and single-stranded nucleotides [10,11]. Affine gap penalties are also used.

The alignment of two sequences *I* and *K* is initiated by aligning one nucleotide from each sequence to another. These are then expanded into longer alignments using dynamic programming. The central part of the recursion can be seen below. A more complete recursion can be seen in the recursion section of Protocol S1.

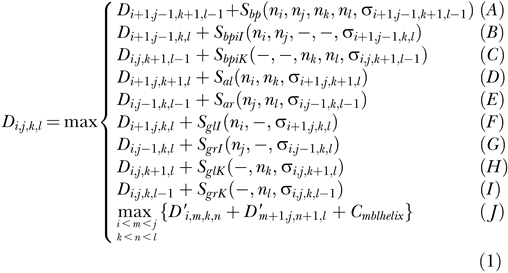



*D_i,j,k,l_* is the score of an alignment of subsequence (*i*,*j*) from sequence *I* to subsequence (*k*,*l*) from sequence *K*. *S_bp_* through *S_grK_* are the costs of adding one or more nucleotides to the alignment. σ is the structure context. *i* & *j*, *k* & *l*, are the start and end coordinates of a subalignment in sequence *I* and *K*, respectively. *n_i_* is the nucleotide at position *i* (in sequence *I*). Likewise for *n_j_, n_k_,* and *n_l_*_._ Here, unpaired nucleotides in branched loops are scored like unpaired nucleotides in external loops. Therefore, the alignment score *D* must be corrected if the context is not the external loop context. The score including this correction is called *D*′, and it is not necessary to store it in memory since it is easily calculated from *D*; see Protocol S1. *C_mblhelix_* is a cost for adding extra stems.

**Figure 1:**
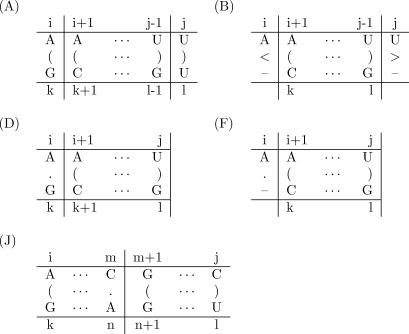
The Cases of Equation 1 (A) Adds a conserved basepair. In the structure, a conserved basepair is indicated using ( and ). (B) Adds aligned unpaired nucleotides. An insert basepair is indicated using < and >. (D) Adds aligned unpaired nucleotides. An unpaired nucleotide is indicated with a “.”. (F) Adds unpaired insert nucleotides. (J) Equation 1J (shown) joins substructures into one structure. Due to the bifurcation constraint, the nucleotide at position *i* must be basepaired to a nucleotide in the subsequence from *I* + 1,...,*m*, and the nucleotides at position *m* + 1 and *j* must also basepair. The same constraints are placed on the corresponding nucleotides in the other sequence. See Figure 2 for extra details.

Equation 1A adds a base pair in both structures. Equations 1B–1C add base-pair inserts in either of the structures. Equations 1D–1E add aligned unpaired nucleotides in either end of the alignment. Equations 1F–1I add an unpaired nucleotide aligned to a gap to the alignment. Equation 1J is the bifurcation case which joins two substructures into one in each of the structures. Figure 1 gives an overview of the different Equation 1 cases.

**Figure 2:**
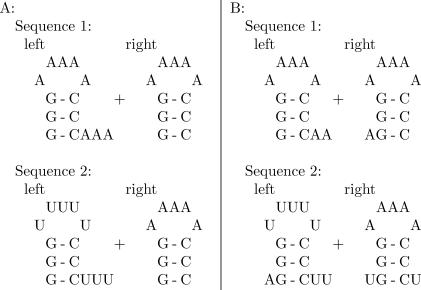
The Bifurcation Constraint (A) The allowed case. The first nucleotides of the left substructures in both sequence 1 and sequence 2 are basepaired, and the first and last nucleotides in the right substructures are basepaired to each other. (B) A disallowed case. The score of the joined alignment will not be calculated using the bifurcation-loop calculation (the resulting alignment will, however, be calculated by expanding the alignment result from (A). There are two reasons: the first nucleotide of the left substructure in sequence 2 is not basepaired, and the first and the last nucleotides of the right substructures are not basepaired in both sequences.

In addition to the new pruning constraint (see below), the algorithm employs three old constraints. 1) The maximum motif length constraint λ limits the maximum length of the subsequences in the resulting alignment (the final alignment can only be longer than λ due to gaps). The use of λ allows the program to split the shortest of the sequences into smaller chunks and scan a λ nucleotide–long window along the other. For details see [11]. 2) The maximum length difference between any two subsequences is constrained to δ nucleotides. These two constraints reduce the time complexity to a maximum of *O*(*L_I_L_K_λ* ^2^ *δ* ^2^) and the memory complexity to a maximum of *O*(*λ* ^3^ *δ)*. L_I_ and L_K_ are the lengths of the sequences. 3) The bifurcation constraint limits the types of substructures that can be joined together in the case of Equation 1J. The constraint requires that the first nucleotide of each of the two (one from each sequence) upstream substructures, *D_i_*_,*m*,*k*,*n*_, are base-paired. Furthermore, the end nucleotides of the downstream structures, *D_m_*_+1,*j*,*n*+1,l_, must form a base pair with each other, see Figure 2. In [11], the bifurcation constraint was used to save time, but here it is also used to save memory. Inspection of Equation 1 shows that the cases A–I only depend on the scores of alignments with either coordinate *i* or coordinate *i* + 1. Case J depends on subalignments with start coordinates *i* and coordinates *i* < *m + 1* < *j*. Due to the bifurcation constraint, all alignments with start coordinate *m + 1* must have the stem context. It is therefore not necessary to keep information about alignments in any other context for coordinates *i* + 2,...,*i* + λ. This saves a large amount of memory, but leads to trouble during backtrack, see below. Further details can be found in the Memory implementation section of Protocol S1.

The new implementation of the FOLDALIGN algorithm has a lower time and memory complexity than the old implementation during global alignment. Since a global alignment must include both ends of both sequences, the δ parameter can be used to also limit the start coordinate of a subalignment in the second sequence. In this way the δ parameter becomes similar to the *M* parameter as used in [35]. The new time complexity is 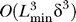 and the memory complexity is 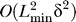 , where *L_min_ = min*{*L_I_, L_K_*}. The old implementation has a time complexity of 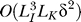 and a memory complexity of 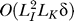 since it used the local-alignment algorithm with λ equal to the sequence lengths.

#### Changes in the energy model.

There are three changes in the energy model compared with [11]. Single-stranded nucleotides, external to all base pairs, are now always scored in the same way, namely as single-stranded nucleotides in multibranched loops; see for example the “Development and References” document at the mfold Web site http://frontend.bioinfo.rpi.edu/zukerm/rna/energy/node2.html#SECTION20 [25,36]. This includes the affine energy cost which is usually not used in the calculation of the energy of external nucleotides. The inclusion of this cost helps keep multibranched and external loops from growing unchecked. See also the release file of the software package. Inserted base pairs are now allowed at all positions in a stem except for the first base pair. The stem must still have at least two conserved base pairs. Single-strand nucleotides in multibranched loops that are next to base-paired nucleotides are no longer stacked, i.e., dangling ends are no longer used. The performance gained by using dangling ends does not justify the complexity that they add to the algorithm.

#### Pruning.

The dynamical programming matrix, *D*, is pruned as it is being filled out by comparing the FOLDALIGN score *D_i,j,k,l_* of a subalignment to a threshold, Θ*_local_*. Thus, for local alignments, if

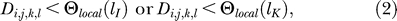

*D_i,j,k,l_* is not stored in the memory. An alignment that has been pruned away cannot be part of any longer alignments, and it is therefore not necessary to take it into account when the longer alignments are calculated. This saves a lot of time. Note that the alignment score is maximized not minimized. For additional details, see the Memory implementation section in Protocol S1. Note that Θ*_local_* depends on the lengths *l_I_* and *l_K_* of the subsequences being aligned. To find the exact length dependency, several schemes were tested, and we found that a simple linear form with Θ_local_ = a * min{*l_I_,l_K_*} *+ b* worked well. The parameters *a* and *b* were found using the dataset containing 99 sequence pairs described in the data section of Materials and Methods. The default values can be seen in Table 1. The linear dependency is not surprising as the alignment score is expected to grow linearly with the alignment length [37].

**Table 1:**
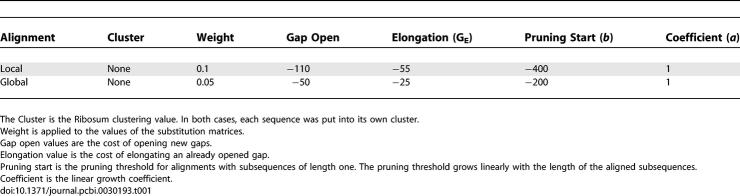
The Parameters of the Default Score Matrices for Local and Global Alignment

Since a global alignment extends over the entire sequence length, it is necessary to insert a minimum number of gaps, equal to the length difference between the sequences. The simple pruning scheme used during local alignment was found to be too strict, pruning away all subalignments in many cases. The following pruning scheme is therefore used during global alignment:

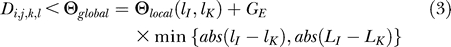



Θ*_global_* is the pruning score used during global alignment. Θ*_local_* is the local-alignment pruning score (Equation 2). *G_E_* is the gap-elongation cost. It should be noted that a new set of *a* and *b* is used. These were found using the tRNA and 5S rRNA datasets described in Materials and Methods. The default values can be seen in Table 1. See further discussion in the Global-Alignment Results section.

#### Backtrack.

When the coordinates of the best-scoring local alignment have been found, the dynamic programming matrix may no longer cover the sequence region of the best hit. The subsequences must therefore be realigned. This realignment is a global alignment of the two subsequences, and the time and memory complexities are therefore the same as for global alignment. While the theoretical memory complexity is lower during backtrack than during local alignment, the actual memory requirement is usually larger. This is because during backtrack it is necessary to trace back through all the cells in the four-dimensional matrix that were passed during the alignment. It is therefore necessary to store information for all subalignments regardless of the basepairing, i.e., the bifurcation constraint cannot be used to save memory. Subalignments that have been pruned can still be pruned since the backtrack cannot pass through a pruned subalignment.

**Figure 3:**
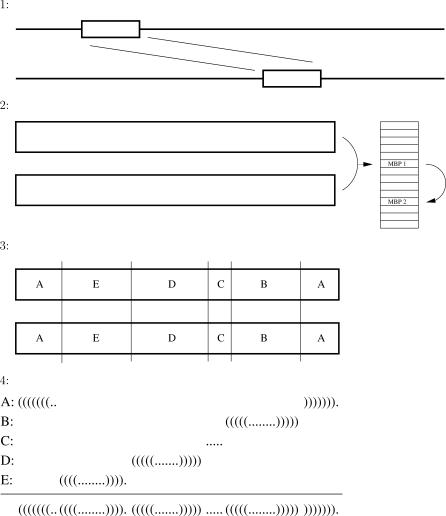
The Divide and Conquer Algorithm (1) In the first step, the region of interest is found using the local-alignment algorithm. During the local alignment, the bifurcation constraint is used to save a lot of memory. (2) In the second step (first step of a global alignment), the region of interest is realigned. The bifurcation constraint is still used, but an additional list of branch points is made. The list stores the six coordinates of the branch point and pointers to the next branch points (one for each of the two substructures). For each subalignment, a pointer to the last branch point is kept. (3) Using the branch point pointers and coordinates, the alignment is split into shorter unbranched segments. Here A is an initial unbranched segment. MBP 1 (Multi Branch Point) splits the alignment into two segments: EDC and B. C is a new initial unbranched segment. MBP 2 splits the ED segment into the E and D segments. (4) The five segments are realigned and backtrack without using the bifurcation constraint to save memory. These realignments are fast, as it is not necessary to evaluate the bifurcation part of the algorithm. Finally, the segments are joined together into the final alignment.

A strategy somewhat similar to the divide and conquer strategy in [38] is used to try to circumvent this problem. The strategy is shown in Figure 3. After the region of interest has been found using a local alignment, the next step is to realign the subsequences. During this realignment, a list of all bifurcation points is saved. For each subalignment, a reference to the last bifurcation point in that given alignment is kept. In addition to all the subalignments with stem context, subalignments with bifurcation context are also stored for *i* + 2,...*i* + λ. This is used to do a pre-backtrack which locates all bifurcation points in the alignment. With all the bifurcation points located, the alignment is divided into a series of hopefully shorter unbranched subalignments. These are then realigned, saving all subalignments for all *i*'s, and backtracked in a normal fashion. The realignment of the unbranched subalignments is fast since it is not necessary to evaluate the bifurcation part of the algorithm. The time complexity of each of these realignments is therefore 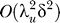 , where *λ_u_* is the length of the unbranched subalignment.

### Local-Alignment Results

**Figure 4:**
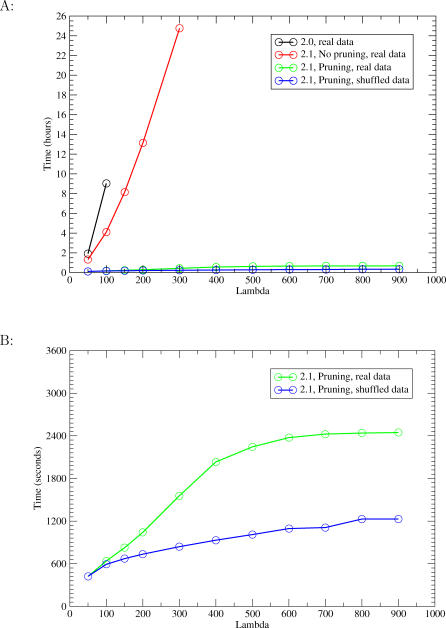
The Average Run Time (A) Time requirements as a function of λ. The “real data” curves were made using a SRP dataset. It contains eight pairs of 1,000 nucleotide-long sequences. Each sequence contains one SRP gene with a length of approximately 300 nucleotides. The “shuffled data” curve was made using shuffled versions of the same dataset. The “2.0” curves were made using the previous version of the program. “No pruning” curves were made using the current version, but without pruning (option -no_pruning). “Pruning” curves were made using the current version and default values of pruning. It is clear that the time needed to make the alignments explodes when pruning is not used, whereas the run time remains much lower when pruning is used. (B) The pruning curves with a smaller time scale. The curve for the sequences containing a motif grows much faster than the curves for the shuffled sequences until the point where λ is as long as the motif. After this, the curves appear to grow at the same rate.

**Figure 5:**
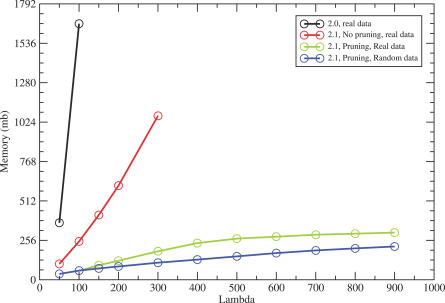
The Average Memory Requirements Memory requirements as a function of λ. See Figure 4 for details.

Figures 4 and 5 show the average time and memory needed to locally align two 1,000 nucleotides–long sequences with different values of λ using different versions of FOLDALIGN. The “real data” curves are for sequence pairs that contain a ∼300 nucleotides–long SRP gene in its genomic context. The “shuffled data” curve is for the same sequence pairs shuffled while conserving the dinucleotide distribution [39]; for details about the dataset see [11]. The curve marked “2.0” is for the previous version of FOLDALIGN. The “No pruning” curve is for the current version using the option -no_pruning which turns off the pruning. The “Pruning” curves are for the current version using the default pruning.

Figure 4 shows that the dynamical pruning has a dramatical impact on the run time of the algorithm. Especially in the case of shuffled sequences where there are no conserved motifs. This is very important during large-scale searches for ncRNAs where a large amount of sequences, many of which do not contain a motif, are aligned [6]. When there is a conserved motif, the speedup is slightly less pronounced, but it is still very drastic. Comparing the “2.0” and the “No pruning” curves, it is also clear that the new implementation is a lot faster even without pruning. The time needed to run the 99 sequence pair dataset used for finding the parameters (pairs of 500 nucleotide–long sequences, see Materials and Methods; λ = 200, δ = 15) dropped from ∼397 CPU hours to ∼9 CPU hours.

Figure 5 shows that pruning also has a significant impact on the required memory. It also shows that it is now possible to align sequences using much larger λ. It is clear from Figure 5 that more memory is required for aligning sequences that contain a conserved structure than for aligning sequences without conserved structures.

The parameters of the algorithm can be split into three groups: Energy, Substitution, and Free. The energy parameters are taken from energy minimization (multiplied by −10 mol/kcal, since we are maximizing a score) [25]; see also the release notes of the software package. The substitution scores are the Ribosum-like log odds scores with one set of scores for single-stranded nucleotides and one for base-paired nucleotides. There are four free parameters: the Ribosum clustering percentage, the gap open, the gap-elongation penalties, and the energy-substitution weight. To simplify the search for the best set of parameters, the gap-elongation parameter was fixed at half the value of the gap-open parameter. The best values for the parameters were found by aligning the 99 sequence dataset using a range of values for each of the free parameters and choosing the values yielding the best performance. The parameters can be seen in Table 1. See Materials and Methods and [11] for details about the data.

To get the background distribution, each sequence was shuffled 20 times conserving the dinucleotide distribution [39] (see Materials and Methods). The extreme value distribution parameters were averaged independently for each of the sequences. The parameters found from the alignment of shuffled sequences were then used to estimate the significance of the alignments from the real sequences pairs. A *p*-value (see Materials and Methods) cutoff of 0.2 was found to be optimal for selecting the significant alignments. The performances (see Materials and Methods) on the 99 sequence dataset can be seen in Table 2. While this method for determining the parameters of the extreme value distribution is biased in several ways, most notably by the use of short sequences compared with the expected length of a random alignment and the finite length effects, the method does seem to yield reasonable results. Fewer than 20 shufflings can be used with little effect on the performance (unpublished data).

**Table 2:**
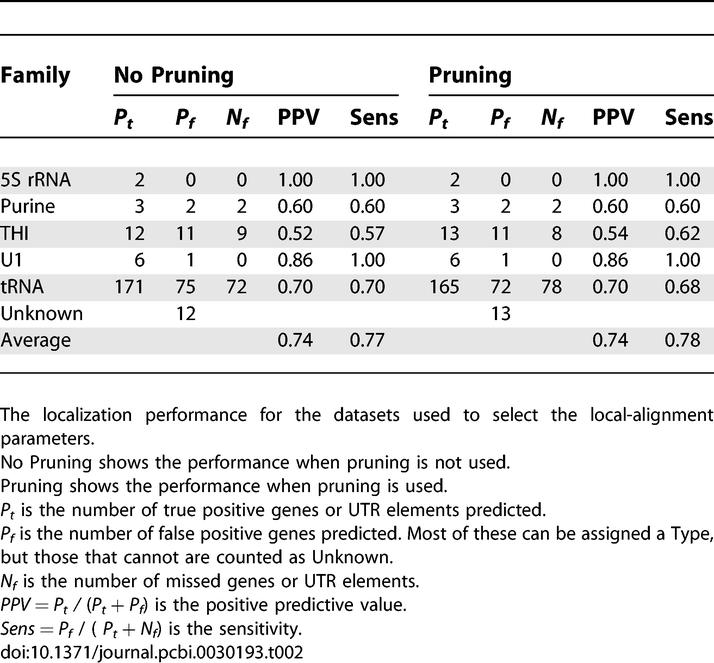
The Localization Performance

The SRP dataset was used as an independent test set. The extreme value distribution parameters were estimated from the alignment of 20 shufflings of each sequence pair and a *p*-value cutoff of 0.2 was used. This yielded eight out of eight SRP genes, and three false positive alignments. The estimated *p*-value of the eight true hits is <0.0005. The *p*-values of the three false positives are <0.0005, 0.069, and 0.134. The positive predictive value, *PPV*, is 0.73, and the sensitivity is 1.00. This shows that the algorithm can be used as a general tool for finding new RNA structures.

### Global-Alignment Results

Initial tests of FOLDALIGN's global-alignment performance while using pruning showed that the simple pruning used during local alignment removes too much during global alignment. Many sequence pairs simply did not produce an alignment. The “problem” is that the global alignment must insert a minimum number of gaps equal to the length difference between the two sequences. When the length difference is large, the cost of inserting the minimum number of gaps is enough to make the algorithm prune away all alignments. To circumvent this problem, a special global-alignment pruning scheme is used; see the previous section called Pruning.

**Figure 6:**
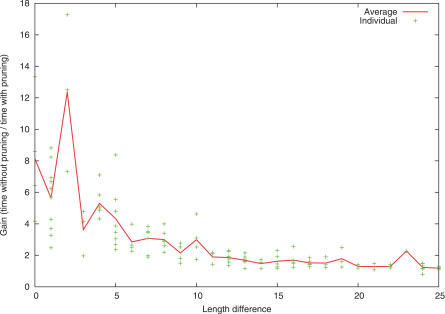
The Time Gained by Using Pruning The global-alignment time without pruning divided by the alignment time with pruning as a function of the length difference between the input sequences. The SRP dataset and δ = 25 was used. The curve shows the average gain. The points are the individual measurements.

Using the global pruning scheme, most global alignments between related sequences produce an alignment. Unfortunately, this also lowers the efficiency of the pruning significantly. Figure 6 shows the time needed to do an alignment without pruning divided by the time needed to do the same alignment using pruning as a function of the length difference between the two sequences. When the length difference is small, it is significantly faster to use pruning. When the length difference is large, there is only a small speed advantage. At a length difference of 25, the use of pruning is only ∼20% faster than not using pruning.

The global 5S rRNA and tRNA datasets (see Material and Methods) were used to select the parameters of the global score matrix, see Table 1 (gap penalties, substitution versus energy weight, and Ribosum clustering percentage). The global SRP and RNaseP datasets were used as validation datasets. As a performance measure, the Matthews correlation coefficient (MCC) of the base-pair prediction was used [40]. Correctly predicted base pairs are counted as true positives, predicted base pairs that are not found in the annotation are counted as false positives. Annotated base pairs not found in the prediction are counted as a false negative. Positions not predicted to base-pair that are not annotated to base-pair, are counted as true negatives. The average MMCs are: 5S rRNA 0.81, tRNA 0.86, RNaseP 0.50, and SRP 0.49. The results for the RNaseP and SRP datasets indicate that the good performance reported for 5S rRNA and tRNA may be due to overfitting. If this were the case, then it should also be possible to overfit on the RNaseP and SRP datasets. These datasets were therefore used to find alternative sets of parameters. The best MCCs found for both datasets were 0.56. The poor performance therefore does not seem to be due to overfitting. Some of the performance difference is likely to be due to structural inserts in the structures. Some of the sequence pairs in both the RNaseP and the SRP datasets contain stem inserts which FOLDALIGN currently cannot handle. The 5S rRNA and tRNA datasets contain fewer large stem inserts.

**Figure 7:**
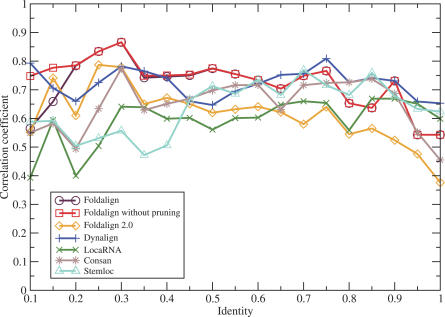
Structure Correlation Coefficient The structure correlation coefficient as a function of sequence identity. The dataset consists of tRNAs and 5S rRNAs; for details see [12]. The difference in correlation coefficient for “FOLDALIGN” and “FOLDALIGN without pruning” at identities 0.1 and 0.15 is due to three sequence pairs for which no alignment and structure are produced. Rerunning FOLDALIGN for those three pairs without pruning should be trivial. “Stemloc” did not produce an alignment for two pairs. For both FOLDALIGN and Stemloc, the pairs which did not produce an alignment are counted as zero.

Recently Dowell and Eddy published results [12] which showed that while FOLDALIGN makes good alignments, its base-pair prediction sensitivity is slightly lower than that of other methods for folding and aligning RNA sequences. Since the dataset used in [12] also contains more sequence pairs than the Bralibase dataset [7], the dataset [12] is used to test the global-structure prediction performance of the algorithm. Figure 7 shows the performance of the old and new versions of FOLDALIGN and some of the other methods which can make pairwise structural alignments of RNA using the dataset from [12]; see Table 3 for details about the methods. In addition to the methods shown in Figure 7 and Table 3, the combination of RNAcast and RNAforester as described in the main page of the RNAshapes package was also tested. The combined method only produced an alignment for less than 60% of the sequence pairs and the results are therefore not reported. In cases where the combined method did produce an alignment, the alignment usually looked good. The data used is the dataset from Figure 7 in [12]. The performance measure is the MCC (see Materials and Methods). Figure 7 shows that the performance has improved from the old to the new version of FOLDALIGN. It also shows that the pruning constraint does not affect the performance in most ranges of sequence identity. Only in the identity range from 0.1 to 0.2 is there a significant difference. This is due to three sequence pairs (less than 1% of the dataset) for which no alignments are found. In these cases, pruning is not feasible; however, an alignment can still be obtained by running FOLDALIGN without pruning. Table 3 shows how long a time it takes to align the full dataset [12]. The memory requirements are also shown in Table 3. Two types of memory consumption are shown. The first (Max) is the maximum amount of memory (Resident Set Size, RSS) used during the alignment of the entire dataset. The second (Ave) is the average amount of memory needed to align each of the pairs. For “FOLDALIGN 2.1” and “FOLDALIGN 2.1 -no pruning”, the Max number (68 Mb and 323 Mb, respectively) are due to a pair of tRNAs which are predicted to a have a stemloop structure. The divide and conquer algorithm can therefore not be used to split the backtrack into smaller sections, and the memory requirement becomes high. Solutions to the stemloop problem can be to use a cubic space models [32,33] or a method similar to the Treeterbi algorithm [34]. If this one alignment is ignored, the Max memory consumptions are 21 Mb and 77 Mb, respectively. Locarna [17] is the fastest and requires the least amount of memory, but it is also the least accurate. FOLDALIGN is slower and uses more memory, but it is also one of the most accurate. Dynalign [24] also makes accurate alignments but is slower than FOLDALIGN. Consan [12] and Stemloc [22] are slow and use large amounts of memory without being more accurate than Dynalign and FOLDALIGN. The computer used to make these tests runs Linux (kernel 2.6) on two 2.4-GHz 32-bit Intel Xeon CPUs, and it has 4 Gb of memory.

**Table 3:**
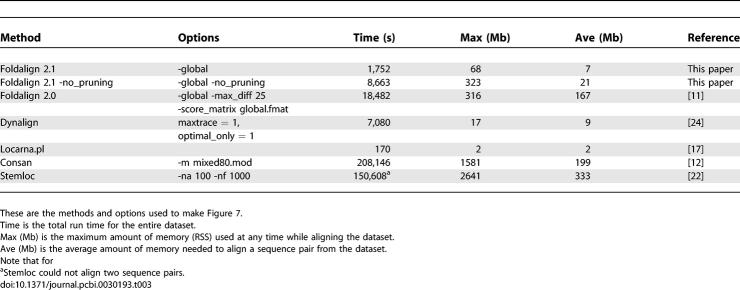
Pairwise Methods for Predicting Structures and Alignments of RNAs

## Discussion

FOLDALIGN is a fast and efficient tool for making pairwise local or global alignments of RNA sequences. Whereas there exist a number of methods able to make global alignments, FOLDALIGN seems still to be one of the very few tools that can do pairwise local structural alignment of RNA sequences. Thus the main motivation was in particular to improve on this aspect of FOLDALIGN. In accordance, this paper described a new way of improving the run time and memory efficiency of dynamical programming methods. Furthermore, there are several improvements to the software package. The main changes are: pruning of the dynamical programming matrix, a better implementation of memory usage, and a better energy model.

The pruning of the dynamical pruning matrix works by requiring that a subalignment must have a score above a length-dependent cutoff. Otherwise, the subalignment is removed from the dynamical programming matrix and cannot be part of any longer subalignment. Pruning efficiently slashes run time and memory requirements without degrading the predictive performance. Using pruning to speed up other dynamical programming applications should be straightforward.

The memory usage of the implementation is further improved in two ways: in the branch point calculation and during backtrack. In the calculation of branch points, two substructures are only added together if the nucleotides at the start and end positions of the downstream substructure are base-paired. Therefore, no downstream subalignment is saved unless its start and end positions base-pair. A similar method was used to speed up the algorithm in [11], but now it is used to both speed up the calculation and to lower the memory consumption. During backtrack, information is needed for every cell in the dynamic programming matrix that will be passed by the traceback algorithm (pruned alignments will not be passed and pruned cells will therefore not be needed). A divide and conquer approach has been implemented which first locates the branch points in the common structure, and then uses them to divide the structure into hopefully smaller unbranched segments. These can then be backtracked separately. While the improvement to the branch point calculation and the divide and conquer approach are more specialized than the pruning heuristic, they are likely to be of use for other RNA alignment methods.

The improvements in speed and memory requirements are important as they make studies like [6] more feasible, and thereby help to elucidate ncRNA genes' and structures' role in molecular biology.

The energy model has been improved in two ways. The first is that insert basepairs are now allowed at any position in a stem except for the first one. The second improvement is that external single-stranded nucleotides are now always scored in a consistent way—namely, as single-stranded nucleotides in a multibranched loop.

Even though the main focus for the FOLDALIGN algorithm is local alignment, the global-alignment test shows that the algorithm is the most accurate method for making global alignments of low-similarity sequences. There are several directions where the resource requirements can be improved upon. Firstly, one could introduce an align-then-fold step, to the aid of finding a lower bound for the pruning threshold. Secondly, a pre-scan for all dinucleotides could be made to initiate all stems. Thirdly, restricting the fold envelope (as introduced by Holmes [22]) by compiling a list of different shapes [19,20] might be used as a further constraint. Finally, adding a Markov model, as in Harmanci et al. [24], for initial restriction of the align envelope might aid in further resource improvements.

The FOLDALIGN software package is released under the GNU public license. It has been tested on Linux operating systems, but should run on any system that can compile standard ANSI C++. Invoking the FOLDALIGN program with the option *-help* will print a short description of all available options. The package can be downloaded from http://foldalign.ku.dk.

In conclusion, FOLDALIGN constitutes an efficient tool for pairwise local and global structural alignment of RNA sequences. With the introduction of pruning, the time of the genomic screen (ten chromosomes between human and mouse) in [6] was reduced from five months (on 70 CPUs) to only one week.

## Materials and Methods

### Data.

Two datasets are used to optimize the local-alignment parameters and evaluate the performance. Each sequence pair in the datasets contains at least one conserved RNA structure and the surrounding context. The sequences of the region with this conserved structure are at most 40% identical. The single-strand minimum folding energy of the conserved structure is indistinguishable from the folding energy of the surrounding sequence. The genomic contexts were found in GenBank [41]. The first dataset (used for optimizing the parameters) consists of 99 sequence pairs. The sequence plus context sequence is 500 nucleotides long. The conserved structures are 5S rRNA (two pairs) [42], Purine riboswitches (five pairs) [43–45], THI riboswitches (21 pairs) [45–50], tRNA (65 pairs) [51], or U1 (six pairs) [52]. For some of the sequences, there are other RNAs annotated within the context region (mainly tRNAs). These extra RNAs are annotated in the dataset if the entire RNA sequence is part of the context. Partial RNAs are annotated as intergenic sequence. The total number of the tRNA pairs in the dataset is therefore 277. The second dataset (used to evaluate the performance) consists of eight SRP pairs [53]. For details about the datasets, see [11].

To train and test FOLDALIGN's global-alignment performance, a new dataset has been made. The sequence pairs of the dataset were selected from the 5S rRNA, RNaseP, SRP, and tRNA databases [42,53–55]. Any sequences containing nucleotides other than *A*, *C*, *G*, or *U* were removed from the databases. A few sequences which obviously did not fit into the databases were removed. Then the sequences in each database were redundancy-reduced to 90% similarity using the Hobohm 2 algorithm [56]. Sequence pairs were selected from the remaining sequences by sorting the pairs by their identity and selecting the pairs with the lowest identity. Each sequence can only be part of one sequence pair. The structures were cleaned by annotating any non *A - U, G - C,* or *G - U* base pair as single-stranded. Nucleotides annotated to base pairs with gaps were also reannotated to be single-stranded.

The 5S rRNA database has three separate sections (Eubacteria, Eukaryota, and Archaea). Each section was treated separately before the final datasets were joined. This part of the data contains 215 sequence pairs. From the RNaseP database, only the sequences in the bacterial type A alignment [57] were used, as this alignment seems to have the most sequences and the best annotation. This dataset has 101 sequence pairs. The SRP dataset contains 121 sequence pairs. The pseudo-knot base pairs were removed from the structures. The tRNA dataset contains 1,810 sequence pairs.

The global-alignment dataset made by [12] contains 324 sequence pairs from the tRNA (184 pairs) and 5S rRNA (140 pairs) families of RFAM [45].

### Significance of local alignments.

By default, FOLDALIGN returns the structure, alignment, and positions of the best-scoring local alignment of a pair of sequences. However, it can also output the score and coordinates of the alignment with the highest score compared with the log of its length for each pair of positions (*i*,*k*) in the two sequences [58]. The list of scores and coordinates are turned into a ranked list of non-overlapping alignments. As described in [11], this is done by the following. 1) Find the alignment with the highest score compared with the logarithm of the sequence lengths. 2) Remove all alignments that overlap the alignment found in step 1. 3. If more alignments are available and desired, go back to step 1.

Ideally, the significance of an alignment is found by comparing its score to a large number of scores from shuffled alignments using the extreme value distribution [59,60]. With a method like FOLDALIGN, alignment of thousands of random sequences is not feasible. In [61,62], it is suggested that from each alignment it is possible to use more than just the score of best alignment to find the parameters of the extreme value distribution.

The parameters *κ* and *Λ* (*Λ* is called *λ* in other texts) of the distribution are found using the method described in [37]:

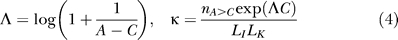
where *A* is the mean of all alignments scoring above a cutoff *C*, and *n_A>C_* is the number of alignments scoring above the cutoff. In [11], several values of *C* were tested, and one assumed to be optimal was chosen. This sometimes led to problems with the distribution being estimated from only one alignment score. We have therefore changed the script to use a fixed value of *C* = 0, which usually yields good results. The probability of getting a random alignment with a score larger than or equal to the score *D* is [37]:

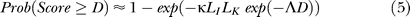



The extreme value distribution parameters are estimated for each sequence pair using alignments of 20 shufflings of that pair. The dinucleotide distribution is conserved during the shuffling [39].

### Local-alignment performance evaluation.

The localization performance is measured by counting the number of structure pairs found (*P_t_*), structure pairs missed (*N_f)_*, and the number of false positive predictions (*P_f_*) made by the method.

The annotated structure pairs overlapped by a prediction are counted as found (*P_t_*) if at least half the nucleotides covered by the prediction in both separate sequences are annotated as RNA structures. If there is more than one *P_t_* prediction which covers the same pair of structures, then the structure pair is only counted once. Predictions in which at least half of the nucleotides are not annotated as RNA in both sequences are counted as false positives (*P_f_*). If a false positive prediction overlaps a structure from one RNA family and only one family in a sequence, then the prediction gets this family, otherwise it gets the “Unknown” family. If a false positive prediction gets an RNA family for one of the sequences and the “Unknown” family for the other, then the false positive is counted as belonging to the known RNA family. A missed structure pair (*N_f_*) is a pair of annotated structures which is not overlapped by any significant positive predictions. Mixed RNA families, like a tRNA versus a 5S rRNA, are ignored. From the *P_t_*, *P_f_*, and *N_f_* numbers, the positive predictive value *PPV = P_t_* / (*P_t_ + P_f_*) and the sensitivity *Sens = P_t_* / (*P_t_ + N_f_*) are calculated.

### Global structure prediction-performance evaluation.

To compare the predicted structures and the annotated structures, the MCC [40] for structures are used:

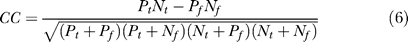



*P_t_* is the number of predicted base pairs which are also annotated. *P_f_* is the number of predicted basepairs which are not annotated. *N_f_* is the number of base pairs that are annotated but not predicted. *N_t_* is the number of positions that are both predicted and annotated not to base pair. As *N_f_* is always very large, the approximation described in [30] could have been used. No correction for sliding base pairs is used [35].

## Supporting Information

Figure S1 State Chart A simplified state chart of the FOLDALIGN energy model. The alignment always starts in the “Start” state which is a hairpin-loop state. The alignment ends in the “End” state. The “External” state recalculates the scores of the “Hairpin-”, “Bulge-”, and “Internal-” loop states to an “External” state score when needed. Unpaired nucleotides in the bifurcation states are scored in the same way as external states. The “Hairpin-loop” state aligns unpaired nucleotides in the hairpin context. The “Stem” state aligns basepairs in both sequences. The “Stem insert” state aligns a basepair in one of the sequences with two gaps in the other. “Bulge right” aligns bulges on the right side of a stem. “Bulge left” aligns bulges on the left side of a stem. The “Internal-loop” state aligns two internal-loops nucleotides. The “Bifurcation” state joins two substructures. The right structure must be in the “Stem” or “Stem insert” state. The state of the left structure must be: “Stem”, “Stem insert”, “Bifurcation”, “Bulge right”, or Bifurcation unpaired right (“Bf. right”). Bifurcation unpaired right aligns unpaired nucleotides on the right side of a branch point. Bifurcation unpaired left & both (“Bf. left & both”) aligns unpaired nucleotides on the left, right, and both sides of a branch point.
(11 KB PDF)

Figure S2 μ *μ* is the number of unpaired nucleotides external to the last basepair. *μ*_1_ is the number of unpaired nucleotides upstream of the last basepair in the first sequence. *μ*_2_ counts the unpaired nucleotides downstream of the last basepair in the first sequence. *μ*_3_ and *μ*_4_ are defined in the same way but for sequence 2. In this example *μ*_1_ *=* 1*, μ*_2_ *=* 2*, μ*_3_ *=* 3*,* and *μ*_4_ *=* 4*.*
(7 KB PDF)

Figure S3 Normal versus Expanding Dynamic Programming A 2-D folding example of standard versus expanding dynamical programming matrices. *i* > *j* are the sequence coordinates.
(A) Is the standard case. The blue cell is filled using the green cells (adding a basepair, or a single stranded nucleotide), and by joining two of the red cells (a bifurcation). Arrows have only been drawn for one of the bifurcations. The grey cells are those which have already been filled.
(B) Is the expanding case. The blue cell is used to partially calculate the score of the green cells (adding a basepair or a single-stranded nucleotide). The yellow cells are the results of joining the blue cell and the red cells in bifurcations. The dark grey cells are those which have already been completely filled. The light grey cells are those where the calculation is not completely finished.
(13 KB PDF)

File Collection S1 FOLDALIGN Software Package (808 KB TAR)

Table S1 Notation (26 KB PDF)

Protocol S1 Supplementary Material (97 KB PDF)

## Abbreviations

MCC: Matthews correlation coefficient
ncRNA: noncoding RNA
