# Supplementary material for: Fast Pairwise Structural RNA Alignments by Pruning of the Dynamical Programming Matrix
Source: PLoS Comput Biol. 2007 Oct 12;3(10):e193. doi: 10.1371/journal.pcbi.0030193 (PMC2014794; doi:10.1371/journal.pcbi.0030193)
Supplement: Supplementary file 1 [file pcbi.0030193.sd002.pdf]

Supplementary material for Fast pairwise local structural RNA alignments by pruning of the dynamical programming matrix

Jakob H. Havgaard[1], Elfar Torarinsson[1,2], Jan Gorodkin[1]

[1] Division of Genetics and Bioinformatic, IBHV, University of Copenhagen, Grønnegårdsvej 3, 1870 Frederiksberg C, Denmark

[2] Department of Natural Sciences, University of Copenhagen, Thorvaldsensvej 40, 1870 Frederiksberg C, Denmark

1 The recursion

This section describes the recursion of the 2.1 version of FOLDALIGN. The recursion as showed here is simplified in two ways. Affine gap penalties are not included, and one basepair long stems are allowed. The notation used in the recursion can be seen in Table S1. Figure S1 shows a simplified overview of the energy model. $D_{(i,j,k,l)}$ is the alignment score, $\sigma_{(i,j,k,l)}$ is the alignment state, $\mu_{1(i,j,k,l)}$, $\mu_{2(i,j,k,l)}$, $\mu_{3(i,j,k,l)}$, and $\mu_{4(i,j,k,l)}$ are the lengths of the single stranded regions external to the last basepair, see Figure S2. S_{bp} to S_{grK} are the scores for adding a set of nucleotides to the alignment.

$$D_{(i,j,k,l)} = \max \begin{cases} D_{(i+1,j-1,k+1,l-1)} + S_{bp}(n_i, n_j, n_k, n_l, \sigma_{(i+1,j-1,k+1,l-1)}) & \text{(a)} \\ D_{(i+1,j-1,k,l)} + S_{bpiI}(n_i, n_j, -, -, \sigma_{(i+1,j-1,k,l)}) & \text{(b)} \\ D_{(i,j,k+1,l-1)} + S_{bpiK}(-, -, n_k, n_l, \sigma_{(i,j,k+1,l-1)}) & \text{(c)} \\ D_{(i+1,j,k+1,l)} + S_{al}(n_i, n_k, \sigma_{(i+1,j,k+1,l)}) & \text{(d)} \\ D_{(i,j-1,k,l-1)} + S_{ar}(n_j, n_l, \sigma_{(i,j-1,k,l-1)}) & \text{(e)} \\ D_{(i+1,j,k,l)} + S_{glI}(n_i, -, \sigma_{(i+1,j,k,l)}) & \text{(f)} \\ D_{(i,j-1,k,l)} + S_{grI}(n_j, -, \sigma_{(i,j-1,k,l)}) & \text{(h)} \\ D_{(i,j,k+1,l)} + S_{glK}(-, n_k, \sigma_{(i,j,k+1,l)}) & \text{(g)} \\ D_{(i,j,k,l-1)} + S_{grK}(-, n_l, \sigma_{(i,j,k,l-1)}) & \text{(i)} \\ \max\limits_{\substack{i<m<j \\ k<n<l}} \{D'_{(i,m,k,n)} + D'_{(m+1,j,n+1,l)} + C_{mblhelix}\} & \text{(j)} \end{cases} \tag{S1}$$

The (a) case adds a basepair in both structures. Case (b) and (c) add basepair inserts in either of the structures. Cases (d) and (e) add aligned unpaired nucleotides in either end of the alignment. Cases (f), (g), (h), and (i) add an unpaired nucleotide aligned to a gap to the alignment. Case (j) joins two substructures into one in each of the structures. The calculation of case (j) is heavily constrained as this case is the $O(N^6)$ part of the algorithm. The equations for calculation the scores S for the different cases are shown in the Contexts subsection.

The calculation is initialized by aligning two nucleotides in the hairpin state: (R_{ss} is the single strand substitution cost for nucleotide n_i and n_k. L_{hp} is hairpin-loop length cost. See Table S1).

$$\begin{gathered} D_{(i,i,k,k)} = R_{ss}(n_i, n_k) + L_{hp}(1,1) \\ \sigma_{(i,i,k,k)} \text{ is set to the hairpin start state} \\ \mu_{1(i,i,k,k)} = 1 \\ \mu_{2(i,i,k,k)} = 0 \\ \mu_{3(i,i,k,k)} = 1 \\ \mu_{4(i,i,k,k)} = 0 \end{gathered} \tag{S2}$$

R_{ss} is the single strand substitution cost for the nucleotides n_i and n_k. L_{hp} is hairpin-loop length cost. See also Table S1.

When the score $D_{(i,j,k,l)}$ is calculated, the score of single stranded nucleotides external to the last basepair and the score of the last basepair may not be correct. It is corrected by this calculation:

$$D'_{(i,j,k,l)} = D_{(i,j,k,l)} + S'(\sigma_{(i,j,k,l)}) \tag{S3}$$

The equation for calculating S' can seen in the Contexts subsection.

C_{mblend}	The cost of closing a multibranched loop
$C_{mblhelix}$	The cost of adding a stem to the multibranch loop (not including the first stem)
C_{mblnuc}	The cost of adding a single stranded nucleotide to a multibranched loop
C_{nGC}	The non-GC stem end cost
D	The score of an alignment. Not corrected for external single stranded nucleotides
D'	The alignment score. Corrected for external single stranded nucleotides
I sequence	One of the two sequences. Usually the longest
K sequence	The other sequence. Usually the shortest
L_{bl}	Bulge length cost
L_{il}	Internal loop length cost. Includes the asymmetry cost.
L_{hp}	Hairpin length cost
μ_1	Length of the single stranded region upstream of the last basepair in the I sequence
μ_2	Length of the single stranded region downstream of the last basepair in the I sequence
μ_3	Length of the single stranded region upstream of the last basepair in the K sequence
μ_4	Length of the single stranded region downstream of the last basepair in the K sequence
R_{bp}	Base pair substitution score
R_{ss}	Single strand substitution score
s	Stacking score
s_{hp}	Hairpin end stacking score
s_{il}	Internal loop end stacking score
σ	The state of an alignment

Table S1: Notation

1.1 Contexts

1.1.1 Base pair (a)

S_{bp} is the cost of adding a basepair to both structures. The S_{bp} score is only calculated if both n_i and n_j basepair, and n_k and n_l basepair. The $\mu_{(i,j,k,l)}$ lengths are set to zero if this case has the highest score in

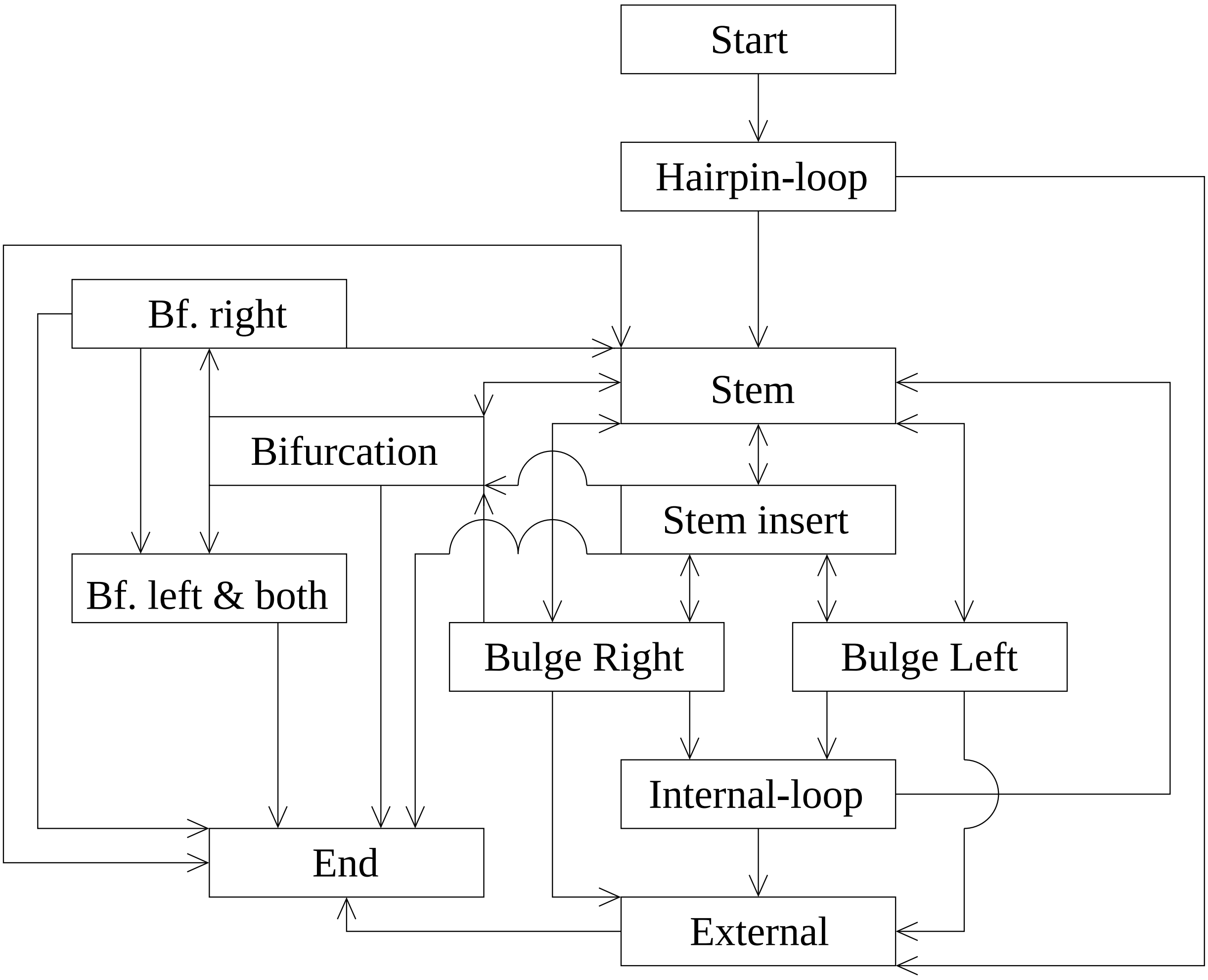

Figure S1: State chart. A simplified state chart of the FOLDALIGN energy model. The alignment always starts in the "Start" state which is a hairpin-loop state. The alignment ends in the "End" state. The "External" state recalculates the scores of the "Hairpin-", "Bulge-", and "Internal-" loop states to an "External" state score when needed. Unpaired nucleotides in the bifurcation states are scored in the same way as external states. The "Hairpin-loop" state aligns unpaired nucleotides in the hairpin context. The "Stem" state aligns basepairs in both sequences. The "Stem insert" state aligns a basepair in one of the sequences with two gaps in the other. "Bulge right" aligns bulges on the right side of a stem. "Bulge left" aligns bulges on the left side of a stem. The "Internal-loop" state aligns two internal-loops nucleotides. The "Bifurcation" state joins two substructures. The right structure must be in the "Stem" or "Stem insert" state. The state of the left structure must be: "Stem", "Stem insert", "Bifurcation", "Bulge right", or Bifurcation unpaired right ("Bf. right"). Bifurcation unpaired right aligns unpaired nucleotides on the right side of a branch point. Bifurcation unpaired left & both ("Bf. left & both") aligns unpaired nucleotides on the left, right, and both sides of a branch point.

```
Sequence 1:    Sequence 2:
   AAA             AAA
 A     A         A     A
   G - C           G - C
   G - C           G - C
 U     UU      UUU     UUUU
```

Figure S2: μ. μ is the number of unpaired nucleotides external to the last basepair. μ_1 is the number of unpaired nucleotides upstream of the last basepair in the first sequence. μ_2 counts the unpaired nucleotides downstream of the last basepair in the first sequence. μ_3 and μ_4 are defined in the same way but for sequence 2. In this example $\mu_1 = 1$, $\mu_2 = 2$, $\mu_3 = 3$, and $\mu_4 = 4$.

equation (S1).

$$S_{bp}(\sigma_{(i+1,j-1,k+1,l-1)}) = \begin{cases} R_{bp}(n_i, n_j, n_k, n_l) + s_{hp}(n_{i+1}, n_{j-1}, n_i, n_j,) + s_{hp}(n_{k+1}, n_{l-1}, n_k, n_l) & \text{if } \sigma \text{ is a hairpin state. } \sigma_{(i,j,k,l)} \text{ becomes a stem state.} \\ R_{bp}(n_i, n_j, n_k, n_l) + s(n_{i+1}, n_{j-1}, n_i, n_j) + s(n_{k+1}, n_{l-1}, n_k, n_l) & \text{if } \sigma \text{ is a stem or an insert basepair state, } \sigma_{(i,j,k,l)} \text{ becomes a stem state.} \\ R_{bp}(n_i, n_j, n_k, n_l) + s(n_{i+\mu_1+1}, n_{j-1}, n_i, n_j) + s(n_{k+\mu_3+1}, n_{l-1}, n_k, n_l) & \text{if } \sigma \text{ is a bulge left state and } \mu_1 \leq 1 \text{ and } \mu_3 \leq 1, \ \sigma_{(i,j,k,l)} \text{ becomes a stem state.} \\ R_{bp}(n_i, n_j, n_k, n_l) + C_{nGC}(n_i, n_j) + C_{nGC}(n_k, n_l) + C_{nGC}(n_{i+\mu_1+1}, n_{j-1}) + C_{nGC}(n_{k+\mu_3+1}, n_{l-1}) & \text{if } \sigma \text{ is a bulge left state and } \mu_1 > 1 \text{ or } \mu_3 > 1, \ \sigma_{(i,j,k,l)} \text{ becomes a stem state.} \\ R_{bp}(n_i, n_j, n_k, n_l) + s(n_{i+1}, n_{j-\mu_2-1}, n_i, n_j) + s(n_{k+1}, n_{l-\mu_4-1}, n_k, n_l) & \text{if } \sigma \text{ is a bulge right state and } \mu_2 \leq 1 \text{ and } \mu_4 \leq 1, \ \sigma_{(i,j,k,l)} \text{ becomes a stem state.} \\ R_{bp}(n_i, n_j, n_k, n_l) + C_{nGC}(n_i, n_j) + C_{nGC}(n_k, n_l) + C_{nGC}(n_{i+1}, n_{j-\mu_2-1}) + C_{nGC}(n_{k+1}, n_{l-\mu_4-1}) & \text{if } \sigma \text{ is a bulge right state and } \mu_2 > 1 \text{ or } \mu_4 > 1, \ \sigma_{(i,j,k,l)} \text{ becomes a stem state.} \\ R_{bp}(n_i, n_j, n_k, n_l) + s_{il}(n_{i+1}, n_{j-1}, n_i, n_j) + s_{il}(n_{i+\mu_1}, n_{j-\mu_2}, n_{i+\mu_1+1}, n_{j-\mu_2-1}) & \text{if } \sigma \text{ is an internal loop state, } \sigma_{(i,j,k,l)} \text{ becomes a stem state.} \\ R_{bp}(n_i, n_j, n_k, n_l) + C_{mblend} + C_{nGC}(n_i, n_j) + C_{nGC}(n_k, n_l) & \text{if } \sigma \text{ is a left or right multibranch loop state, } \sigma_{(i,j,k,l)} \text{ becomes a stem state.} \end{cases}$$

1.1.2 Insert basepair (b) & (c)

S_{bpiI} is a score of inserting a basepair into a stem from the I sequence. The S_{bpiI} score is only calculated when n_i and n_j basepair, and the state is stem or insert basepair in the I sequence. The $\mu_{(i,j,k,l)}$ lengths are all set to zero if this case has the highest score in equation (S1).

$$S_{bpiI}(\sigma_{(i+1,j-1,k,l)}) = 2 \times R_{ss}(gap) + s(n_{i+1}, n_{j-1}, n_i, n_j) \quad \sigma_{(i,j,k,l)} \text{ becomes an insert base pair in the } I \text{ sequence state.}$$

S_{bpiK} is a score of inserting a basepair into a stem from the K sequence. The S_{bpiK} score is only calculated when n_k and n_l basepair, and the state is stem or insert basepair in the K sequence. The $\mu_{(i,j,k,l)}$ lengths are all set to zero if this case has the highest score in equation (S1).

$$S_{bpiK}(\sigma_{(i,j,k+1,l-1)}) = 2 \times R_{ss}(gap) + s(n_{k+1}, n_{l-1}, n_k, n_l) \quad \sigma_{(i,j,k,l)} \text{ becomes an insert base pair in the } K \text{ sequence state.}$$

1.1.3 Align left (d)

S_{al} is score of aligning two single stranded nucleotides on the left side of an alignment.

$$S_{al}(\sigma_{(i+1,j,k+1,l)}) = \begin{cases} R_{ss}(n_i, n_k) & \text{if } \sigma \text{ is a hairpin state,} \\ \quad - L_{hp}(\mu_1) - L_{hp}(\mu_3) & \sigma_{(i,j,k,l)} \text{ becomes a} \\ \quad + L_{hp}(\mu_1 + 1) + L_{hp}(\mu_3 + 1) & \quad \text{hairpin state.} \\ & \\ R_{ss}(n_i, n_k) & \text{if } \sigma \text{ is a stem state or an} \\ \quad + 2 \times L_{bl}(1) & \quad \text{insert basepair state,} \\ & \sigma_{(i,j,k,l)} \text{ becomes a} \\ & \quad \text{bulge left state.} \\ R_{ss}(n_i, n_k) & \text{if } \sigma \text{ is a bulge left state,} \\ \quad - L_{bl}(\mu_1) - L_{bl}(\mu_3) & \sigma_{(i,j,k,l)} \text{ becomes a} \\ \quad + L_{bl}(\mu_1 + 1) + L_{bl}(\mu_3 + 1) & \quad \text{bulge left state.} \\ & \\ R_{ss}(n_i, n_k) & \text{if } \sigma \text{ is a bulge right state,} \\ \quad - L_{bl}(\mu_2) - L_{bl}(\mu_4) & \sigma_{(i,j,k,l)} \text{ becomes an} \\ \quad + L_{il}(1, \mu_2) + L_{il}(1, \mu_4) & \quad \text{internal loop state.} \\ & \\ R_{ss}(n_i, n_k) & \text{if } \sigma \text{ is an internal loop state,} \\ \quad - L_{il}(\mu_1, \mu_2) - L_{il}(\mu_3, \mu_4) & \sigma_{(i,j,k,l)} \text{ becomes an} \\ \quad + L_{il}(\mu_1 + 1, \mu_2) & \quad \text{internal loop state.} \\ \quad + L_{il}(\mu_3 + 1, \mu_4) & \\ & \\ R_{ss}(n_i, n_k) + 2 \times C_{mblnuc} & \text{if } \sigma \text{ is a left or right} \\ & \quad \text{multibranch loop state,} \\ & \sigma_{(i,j,k,l)} \text{ becomes a} \\ & \quad \text{left multibranch loop state.} \end{cases}$$

If this case wins in equation (S1), the lengths are updated like this:

$$\begin{aligned} \mu_{1(i,j,k,l)} &= \mu_{1(i+1,j,k+1,l)} + 1 \\ \mu_{2(i,j,k,l)} &= \mu_{2(i+1,j,k+1,l)} \\ \mu_{3(i,j,k,l)} &= \mu_{3(i+1,j,k+1,l)} + 1 \\ \mu_{4(i,j,k,l)} &= \mu_{4(i+1,j,k+1,l)} \end{aligned}$$

1.1.4 Align right (e)

S_{ar} is the cost of aligning two single stranded nucleotides on the right side of an alignment.

$$S_{ar}(\sigma_{(i,j-1,k,l-1)}) = \begin{cases} R_{ss}(n_j, n_l) & \text{if } \sigma \text{ is a hairpin state,} \\ \quad - L_{hp}(\mu_1) - L_{hp}(\mu_3) & \sigma_{(i,j,k,l)} \text{ becomes a} \\ \quad + L_{hp}(\mu_1 + 1) + L_{hp}(\mu_3 + 1) & \text{hairpin state.} \\ \\ R_{ss}(n_j, n_l) & \text{if } \sigma \text{ is a stem state} \\ \quad + 2 \times L_{bl}(1) & \text{or an insert basepair state,} \\ & \sigma_{(i,j,k,l)} \text{ becomes a bulge right state.} \\ R_{ss}(n_j, n_l) & \text{if } \sigma \text{ is a bulge left state,} \\ \quad - L_{bl}(\mu_1) - L_{bl}(\mu_3) & \sigma_{(i,j,k,l)} \text{ becomes an internal loop state.} \\ \quad + L_{il}(\mu_1, 1) + L_{il}(\mu_3, 1) & \\ \\ R_{ss}(n_j, n_l) & \text{if } \sigma \text{ is a bulge right state,} \\ \quad - L_{bl}(\mu_2) - L_{bl}(\mu_4) & \sigma_{(i,j,k,l)} \text{ becomes a bulge right state.} \\ \quad + L_{bl}(\mu_2 + 1) + L_{bl}(\mu_4 + 1) & \\ \\ R_{ss}(n_j, n_l) & \text{if } \sigma \text{ is an internal loop state,} \\ \quad - L_{il}(\mu_1, \mu_2) - L_{il}(\mu_3, \mu_4) & \sigma_{(i,j,k,l)} \text{ becomes an} \\ \quad + L_{il}(\mu_1, \mu_2 + 1) & \text{internal loop state.} \\ \quad + L_{il}(\mu_3, \mu_4 + 1) & \\ \\ R_{ss}(n_j, n_l) + 2 \times C_{mblnuc} & \text{if } \sigma \text{ is a left multibranch loop state,} \\ & \sigma_{(i,j,k,l)} \text{ becomes a left} \\ & \text{multibranch loop state.} \\ R_{ss}(n_j, n_l) + 2 \times C_{mblnuc} & \text{if } \sigma \text{ is a right multibranch loop state,} \\ & \sigma_{(i,j,k,l)} \text{ becomes a right} \\ & \text{multibranch loop state.} \end{cases}$$

If this case wins in equation (S1), the lengths are updated like this:

$$\begin{aligned} \mu_{1(i,j,k,l)} &= \mu_{1(i,j-1,k,l-1)} \\ \mu_{2(i,j,k,l)} &= \mu_{2(i,j-1,k,l-1)} + 1 \\ \mu_{3(i,j,k,l)} &= \mu_{3(i,j-1,k,l-1)} \\ \mu_{4(i,j,k,l)} &= \mu_{4(i,j-1,k,l-1)} + 1 \end{aligned}$$

1.1.5 Gap left I (f)

S_{glI} is the cost of extending an alignment with one single stranded nucleotide on the left side of the I sequence.

$$S_{glI}(\sigma_{(i+1,j,k,l)}) = \begin{cases} R_{ss}(gap) & \text{if } \sigma \text{ is a hairpin state,} \\ \quad - L_{hp}(\mu_1) + L_{hp}(\mu_1+1) & \sigma_{(i,j,k,l)} \text{ becomes a hairpin state.} \\ & \\ R_{ss}(gap) & \text{if } \sigma \text{ is a stem state} \\ \quad + L_{bl}(1) + L_{bl}(0) & \quad \text{or an insert basepair state,} \\ & \sigma_{(i,j,k,l)} \text{ becomes a bulge left state.} \\ R_{ss}(gap) & \text{if } \sigma \text{ is a bulge left state,} \\ \quad - L_{bl}(\mu_1) - L_{bl}(\mu_1+1) & \sigma_{(i,j,k,l)} \text{ becomes a bulge left state.} \\ & \\ R_{ss}(gap) & \text{if } \sigma \text{ is a bulge right state,} \\ \quad - L_{bl}(\mu_2) - L_{bl}(\mu_4) & \sigma_{(i,j,k,l)} \text{ becomes an internal loop state.} \\ \quad + L_{il}(1,\mu_2) + L_{il}(0,\mu_4) & \\ & \\ R_{ss}(gap) & \text{if } \sigma \text{ is an internal loop state,} \\ \quad - L_{il}(\mu_1,\mu_2) & \sigma_{(i,j,k,l)} \text{ becomes an internal loop state.} \\ \quad + L_{il}(\mu_1+1,\mu_2) & \\ & \\ R_{ss}(gap) + C_{mblnuc} & \text{if } \sigma \text{ is a left or right multibranch} \\ & \quad \text{loop state,} \\ & \sigma_{(i,j,k,l)} \text{ becomes a left} \\ & \quad \text{multibranch loop state.} \end{cases}$$

If this case wins in equation (S1), the lengths are updated like this:

$$\begin{aligned} \mu_{1(i,j,k,l)} &= \mu_{1(i+1,j,k,l)} + 1 \\ \mu_{2(i,j,k,l)} &= \mu_{2(i+1,j,k,l)} \\ \mu_{3(i,j,k,l)} &= \mu_{3(i+1,j,k,l)} \\ \mu_{4(i,j,k,l)} &= \mu_{4(i+1,j,k,l)} \end{aligned}$$

1.1.6 Gap left K (g)

S_{glK} is the cost of extending an alignment with one single stranded nucleotide on the left side of the K sequence.

$$S_{glK}(\sigma_{(i,j,k+1,l)}) = \begin{cases} R_{ss}(gap) & \text{if } \sigma \text{ is a hairpin state,} \\ \quad - L_{hp}(\mu_3) + L_{hp}(\mu_3+1) & \sigma_{(i,j,k,l)} \text{ becomes a hairpin state.} \\ & \\ R_{ss}(gap) & \text{if } \sigma \text{ is a stem state} \\ \quad + L_{bl}(0) + L_{bl}(1) & \quad \text{or an insert basepair state,} \\ & \sigma_{(i,j,k,l)} \text{ becomes a bulge left state.} \\ R_{ss}(gap) & \text{if } \sigma \text{ is a bulge left state,} \\ \quad - L_{bl}(\mu_3) + L_{bl}(\mu_3+1) & \sigma_{(i,j,k,l)} \text{ becomes a bulge left state.} \\ & \\ R_{ss}(gap) & \text{if } \sigma \text{ is a bulge right state,} \\ \quad - L_{bl}(\mu_2) - L_{bl}(\mu_4) & \sigma_{(i,j,k,l)} \text{ becomes an internal loop state.} \\ \quad + L_{il}(0,\mu_2) + L_{il}(1,\mu_4) & \\ & \\ R_{ss}(gap) & \text{if } \sigma \text{ is an internal loop state,} \\ \quad - L_{il}(\mu_3,\mu_4) & \sigma_{(i,j,k,l)} \text{ becomes an internal loop state.} \\ \quad + L_{il}(\mu_3+1,\mu_4) & \\ & \\ R_{ss}(gap) + C_{mblnuc} & \text{if } \sigma \text{ is a left or right multibranch} \\ & \quad \text{loop state,} \\ & \sigma_{(i,j,k,l)} \text{ becomes a left multibranch} \\ & \quad \text{loop state.} \end{cases}$$

If this case wins in equation (S1), the lengths are updated like this:

$$\begin{aligned}
\mu_{1(i,j,k,l)} &= \mu_{1(i,j,k+1,l)} \\
\mu_{2(i,j,k,l)} &= \mu_{2(i,j,k+1,l)} \\
\mu_{3(i,j,k,l)} &= \mu_{3(i,j,k+1,l)} + 1 \\
\mu_{4(i,j,k,l)} &= \mu_{4(i,j,k+1,l)}
\end{aligned}$$

1.1.7 Gap right I (h)

S_{grI} is the cost of extending an alignment with one single stranded nucleotide on the right side of the I sequence.

$$S_{grI}(\sigma_{(i,j-1,k,l)}) = \begin{cases}
R_{ss}(gap) & \text{if } \sigma \text{ is a hairpin state,} \\
\quad - L_{hp}(\mu_1) + L_{hp}(\mu_1 + 1) & \sigma_{(i,j,k,l)} \text{ becomes a hairpin state.} \\[1em]
R_{ss}(gap) & \text{if } \sigma \text{ is a stem state} \\
\quad + L_{bl}(1) + L_{bl}(0) & \quad \text{or an insert basepair state,} \\
& \sigma_{(i,j,k,l)} \text{ becomes a bulge right state.} \\
R_{ss}(gap) & \text{if } \sigma \text{ is a bulge left state,} \\
\quad - L_{bl}(\mu_1) - L_{bl}(\mu_3) & \sigma_{(i,j,k,l)} \text{ becomes an internal loop state.} \\
\quad + L_{il}(\mu_1, 1) + L_{il}(\mu_3, 0) & \\[1em]
R_{ss}(gap) & \text{if } \sigma \text{ is a bulge right state,} \\
\quad - L_{bl}(\mu_2) + L_{bl}(\mu_2 + 1) & \sigma_{(i,j,k,l)} \text{ becomes a bulge right state.} \\[1em]
R_{ss}(gap) & \text{if } \sigma \text{ is an internal loop state,} \\
\quad - L_{il}(\mu_1, \mu_2) & \sigma_{(i,j,k,l)} \text{ becomes an internal loop state.} \\
\quad + L_{il}(\mu_1, \mu_2 + 1) & \\[1em]
R_{ss}(gap) + C_{mblnuc} & \text{if } \sigma \text{ is a left multibranch loop state,} \\
& \sigma_{(i,j,k,l)} \text{ becomes a left multibranch} \\
& \quad \text{loop state.} \\
R_{ss}(gap) + C_{mblnuc} & \text{if } \sigma \text{ is a right multibranch loop state,} \\
& \sigma_{(i,j,k,l)} \text{ becomes a right multibranch} \\
& \quad \text{loop state.}
\end{cases}$$

If this case wins in equation (S1), the lengths are updated like this:

$$\begin{aligned}
\mu_{1(i,j,k,l)} &= \mu_{1(i,j-1,k,l)} \\
\mu_{2(i,j,k,l)} &= \mu_{2(i,j-1,k,l)} + 1 \\
\mu_{3(i,j,k,l)} &= \mu_{3(i,j-1,k,l)} \\
\mu_{4(i,j,k,l)} &= \mu_{4(i,j-1,k,l)}
\end{aligned}$$

1.1.8 Gap right K (i)

S_{grK} is the cost of extending an alignment with one single stranded nucleotide on the right side of the K sequence.

$$
S_{grK}(\sigma_{(i,j,k,l-1)}) = \begin{cases}
R_{ss}(gap) & \text{if } \sigma \text{ is a hairpin state,} \\
\quad - L_{hp}(\mu_3) + L_{hp}(\mu_3 + 1) & \sigma_{(i,j,k,l)} \text{ becomes a hairpin state.} \\[1em]
R_{ss}(gap) & \text{if } \sigma \text{ is a stem state} \\
\quad + L_{bl}(0) + L_{bl}(1) & \quad \text{or an insert basepair state,} \\
 & \sigma_{(i,j,k,l)} \text{ becomes a bulge right state.} \\
R_{ss}(gap) & \text{if } \sigma \text{ is a bulge left state,} \\
\quad - L_{bl}(\mu_1) - L_{bl}(\mu_3) & \sigma_{(i,j,k,l)} \text{ becomes an internal loop state.} \\
\quad + L_{il}(\mu_1, 0) + L_{il}(\mu_3, 1) & \\[1em]
R_{ss}(gap) & \text{if } \sigma \text{ is a bulge right state,} \\
\quad - L_{bl}(\mu_4) + L_{bl}(\mu_4 + 1) & \sigma_{(i,j,k,l)} \text{ becomes a bulge right state.} \\[1em]
R_{ss}(gap) & \text{if } \sigma \text{ is an internal loop state,} \\
\quad - L_{il}(\mu_3, \mu_4) & \sigma_{(i,j,k,l)} \text{ becomes an internal loop state.} \\
\quad + L_{il}(\mu_3, \mu_4 + 1) & \\[1em]
R_{ss}(gap) + C_{mblnuc} & \text{if } \sigma \text{ is a left multibranch loop state,} \\
 & \sigma_{(i,j,k,l)} \text{ becomes a left multibranch} \\
 & \quad \text{loop state.} \\
R_{ss}(gap) + C_{mblnuc} & \text{if } \sigma \text{ is a right multibranch loop state,} \\
 & \sigma_{(i,j,k,l)} \text{ becomes a right multibranch} \\
 & \quad \text{loop state.}
\end{cases}
$$

If this case wins in equation (S1), the lengths are updated like this:

$$
\begin{aligned}
\mu_{1(i,j,k,l)} &= \mu_{1(i,j,k,l-1)} \\
\mu_{2(i,j,k,l)} &= \mu_{2(i,j,k,l-1)} \\
\mu_{3(i,j,k,l)} &= \mu_{3(i,j,k,l-1)} \\
\mu_{4(i,j,k,l)} &= \mu_{4(i,j,k,l-1)} + 1
\end{aligned}
$$

1.1.9 Multibranched loops (j)

Join two alignments to get a multibranched structure. In equation S1 case (j) is only calculated when $\sigma_{(i,m,k,n)}$ is a stem, basepair insert, bulge right, or a right multibranch loop state, and $\sigma_{(m+1,j,n+1,l)}$ is a stem or a basepair insert state. $\sigma_{(i,j,k,l)}$ becomes a right multibranch loop state and the $\mu_{i,j,k,l}$ lengths are set to zero if this case has the highest alignment score. $D_{i,j,k,l}$ is not used directly in this calculation. It is always the external loop version $D'_{ij,kl}$ which is used, see the External nucleotides section below.

1.1.10 External nucleotides

Single stranded nucleotides external to all basepairs must be scored like single stranded nucleotides in multibranched loops. The score must therefore be recalculated when the alignment state is one of the hairpin, bulge, or internal loop states. Furthermore, the cost for non-GC base pairs must also be added in cases where the alignment state is one of the basepair states. This calculation does not affect the state or the μ lengths of the alignment.

$$S'(\sigma_{(i,j,k,l)}) = \begin{cases} (\mu_1 + \mu_3) \times C_{mblnuc} - L_{hp}(\mu_1) - L_{hp}(\mu_3) & \text{if } \sigma \text{ is a hairpin state.} \\ C_{nGC}(n_i, n_j) + C_{nGC}(n_k, n_l) & \text{if } \sigma \text{ is a stem or} \\ & \quad \text{a basepair insert state.} \\ C_{nGC}(n_{i+\mu_1}, n_j) + C_{nGC}(n_{k+\mu_3}, n_l) & \text{if } \sigma \text{ is a bulge left state.} \\ \quad + (\mu_1 + \mu_3) \times C_{mblnuc} - L_{bl}(\mu_1) - L_{bl}(\mu_3) & \\ C_{nGC}(n_i, n_{j-\mu_2}) + C_{nGC}(n_k, n_{l-\mu_4}) & \text{if } \sigma \text{ is a bulge right state.} \\ \quad + (\mu_2 + \mu_4) \times C_{mblnuc} - L_{bl}(\mu_2) - L_{bl}(\mu_4) & \\ C_{nGC}(n_{i+\mu_1}, n_{j-\mu_2}) + C_{nGC}(n_{k+\mu_3}, n_{l-\mu_4}) & \text{if } \sigma \text{ is an internal loop state.} \\ \quad + (\mu_1 + \mu_2 + \mu_3 + \mu_4) \times C_{mblnuc} & \\ \quad - L_{il}(\mu_1, \mu_2) - L_{il}(\mu_3, \mu_4) & \\ 0 & \text{if } \sigma \text{ is a right or left} \\ & \quad \text{multibranch state} \end{cases}$$

2 Implementation of the recursion

The changes to the dynamic programming method described here are not necessary for the use of the pruning heuristic. The changes were only made as they ease the implementation of the FOLDALIGN algorithm.

Traditionally recursions as the one in equation (S1) are implemented just as they are written. To fill out the cell $D_{i,j,k,l}$ the scores of the cells $D_{i+1,j-1,k+1,l-1}$, $D_{i+1,j-1,k,l}$ ... are used. In the current implementation a slightly different approach is used. Here an alignment score $D_{i,j,k,l}$ is expanded into the alignment cells $D_{i-1,j+1,k-1,l+1}$, $D_{i-1,j+1,k,l}$ .... See Figure S3 for an illustration. Rather than taking the maximum of all the cases in equation (S1) at once the maximum must be taken every time a score has been calculated. The reason for this trivial change to the dynamic programming is that the calculation of part (j) of equation (S1) becomes simple to implement. The left part of the bifurcation structure is the $D_{i,j,k,l}$ cell. The right part are all the cells which have start coordinates $i' = j + 1$ and $k' = k + 1$. The implementation simply loops over any cell which starts with these coordinates and meets the constraints (λ, δ, and bifurcation). The bifurcated alignments calculated this way all have the start coordinates (i, k), but the lengths are different.

3 Memory implementation

In addition to making the algorithm much faster the pruning constraint makes it possible run the alignment using much less memory. The bifurcation constraint is also used to lower the memory consumption. The implementation as described below allows the FOLDALIGN algorithm to exploit the lower memory requirements of the pruning heuristic. This is not the only possible way the dynamic programming matrix can be implemented when the pruning heuristic is used. To use the heuristic in another method different implementations should be considered.

A:

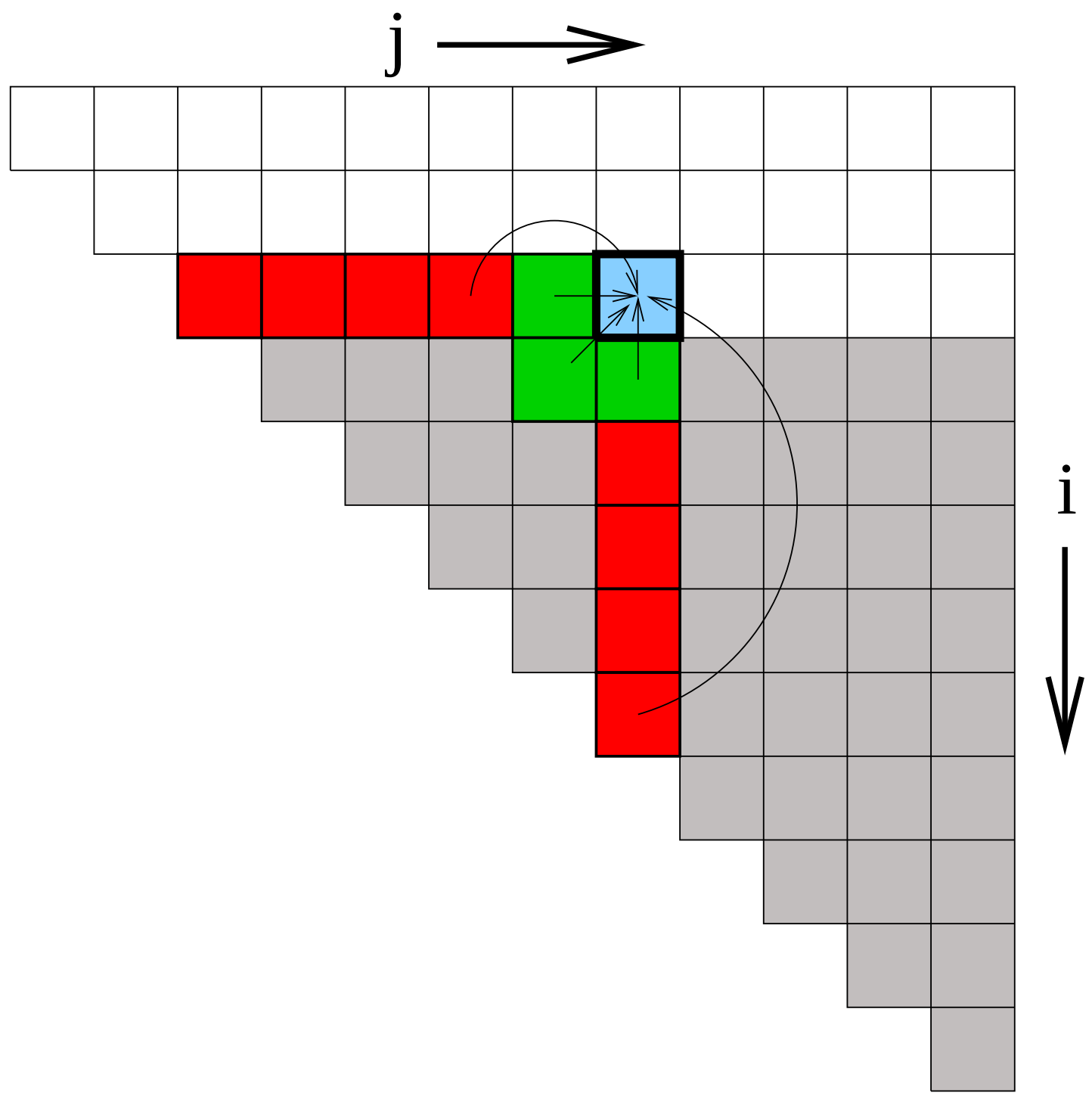

B:

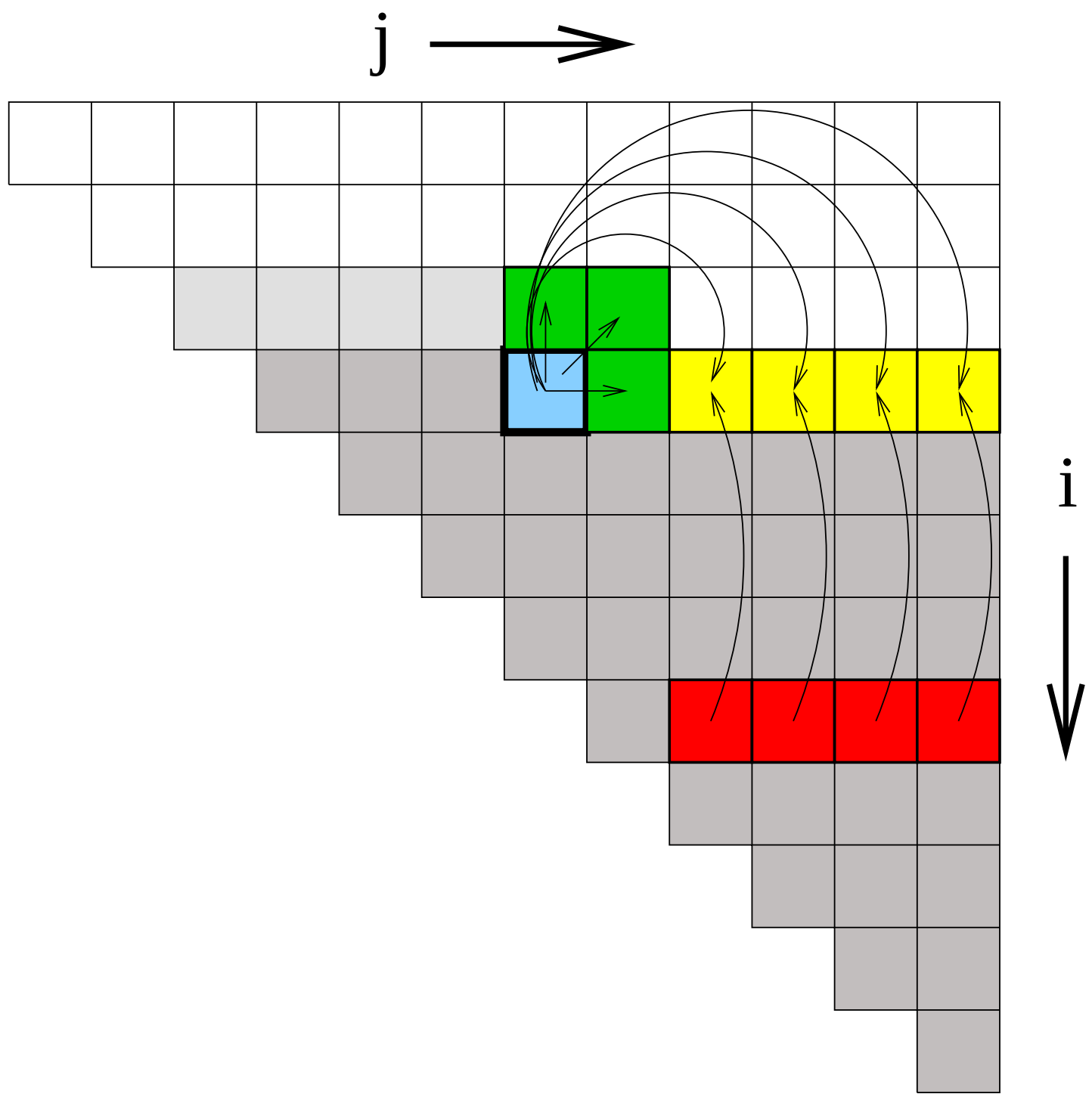

Figure S3: Normal vs. Expanding dynamic programming. A two dimensional folding example of standard vs. expanding dynamical programming matrices. $i > j$ are the sequence coordinates. Case A: is the standard case. The blue cell is filled using the green cells (adding a basepair, or a single stranded nucleotide), and by joining two of the red cells (a bifurcation). Arrows have only been drawn for one of the bifurcations. The grey cells are those which have already been filled. Case B: is the expanding case. The blue cell is used to partially calculate the score of the green cells (adding a basepair or a single stranded nucleotide). The yellow cells are the results of joining the blue cell and the red cells in bifurcations. The dark grey cells are those which have already been completely filled. The light grey cells are those where the calculation is not completely finished.

The pruning constraint saves memory since it is not necessary to store all the information about alignments which have a score below the pruning threshold. The bifurcation constraint saves memory by limiting the number of positions for which it is necessary to store all information about all non-pruned alignments. The bifurcation constraint limits the alignments which must be stored for positions $i' = \{i+2, ..., i+\lambda\}$ to those where the first and the last nucleotides in the subalignment are basepaired to each other. Subalignments where these positions do not basepair are not needed, and are therefore not stored. The dynamic programming matrix is therefore split into two matrices. The short term memory (STM) matrix and the long term memory (LTM) matrix.

The STM matrix stores the alignment score, state, and the four lengths ($\mu_1, \mu_2, \mu_3, \mu_4$, see section 1). As described it is only necessary to hold values for positions i and $i+1$. Therefore the complexity is $O(\lambda^2\delta)$ ($O(\lambda\delta^2)$ for global alignment). The way recursion is implemented the algorithm needs to write to the memory in an unordered fashion due to the bifurcation calculation, see above. The STM matrix is therefore implemented as a 3-dimensional random access array where the last dimension is allocated when needed.

The LTM matrix potentially contains values for all allowed values of i. Therefore the complexity is $O(\lambda^3\delta)$ ($O(\lambda^2\delta^2)$ for global alignment). In this matrix it is only necessary to store the score. The state and lengths are not needed, see the recursion section. The matrix is much more sparse than the STM matrix. It is therefore implemented as a 2-dimensional matrix (i, k) with dimension $O(\lambda^2)$. Each cell in this matrix holds a linked list. Each entry in the linked list holds a window size $W_i = j - i$ and another linked list. The final linked list holds the window size from the other sequence $W_k = l - k$, and the alignment score. Both types of linked list are sorted by the window size.
