# Supplementary material for: Fast Pairwise Structural RNA Alignments by Pruning of the Dynamical Programming Matrix
Source: PLoS Comput Biol. 2007 Oct 12;3(10):e193. doi: 10.1371/journal.pcbi.0030193 (PMC2014794; doi:10.1371/journal.pcbi.0030193)
Supplement: Supplementary file 2 [file pcbi.0030193.sg001.pdf]

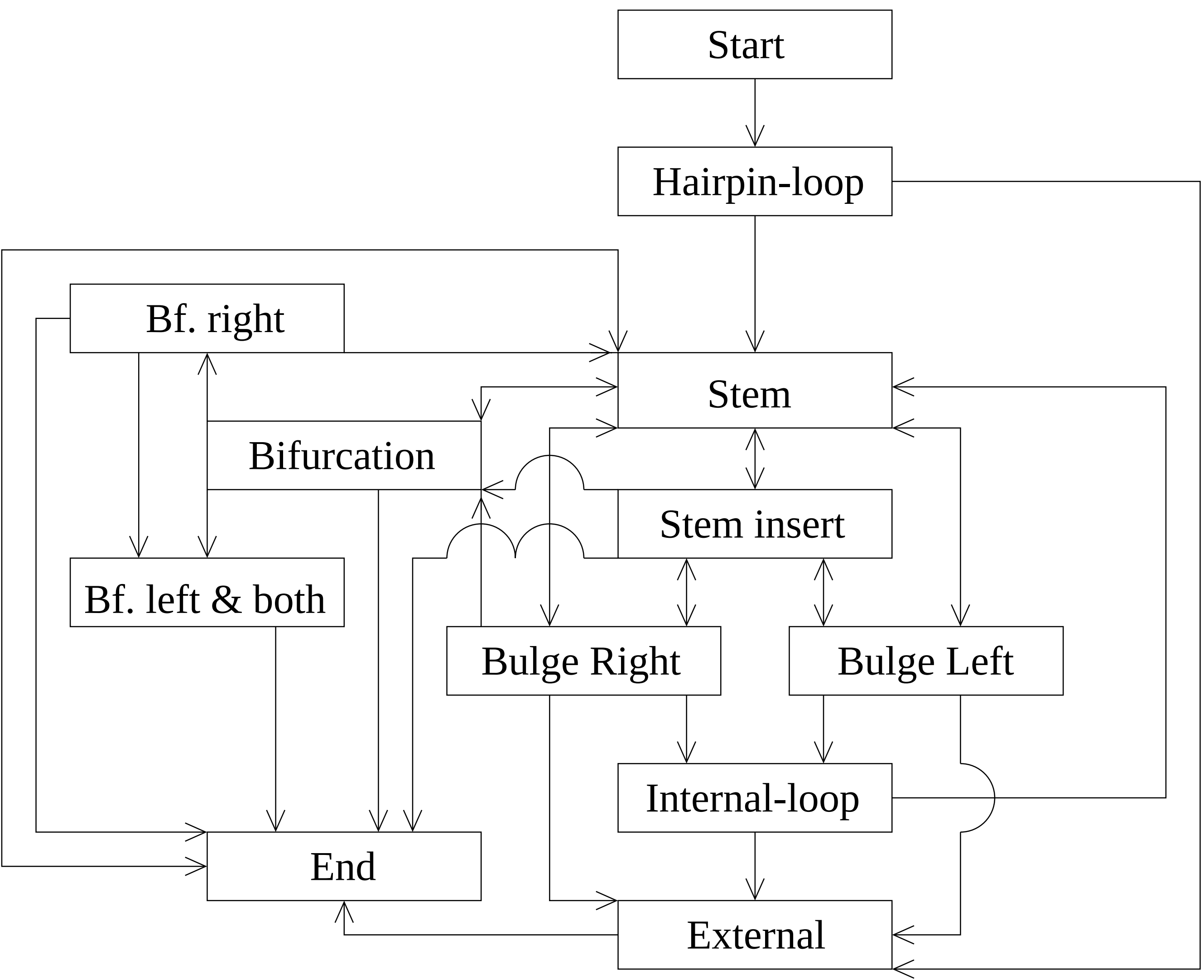
Start
Hairpin-loop
Bf. right
Stem
Bifurcation
Stem insert
Bf. left & both
Bulge Right
Bulge Left
Internal-loop
End
External
