# Supplementary material for: Fast Pairwise Structural RNA Alignments by Pruning of the Dynamical Programming Matrix
Source: PLoS Comput Biol. 2007 Oct 12;3(10):e193. doi: 10.1371/journal.pcbi.0030193 (PMC2014794; doi:10.1371/journal.pcbi.0030193)
Supplement: Supplementary file 3 [file pcbi.0030193.sg002.pdf]

Sequence 1:	Sequence 2:
AAA	AAA
A A	A A
G - C	G - C
G - C	G - C
U UU	UUU UUUU
