# Supplementary material for: Fast Pairwise Structural RNA Alignments by Pruning of the Dynamical Programming Matrix
Source: PLoS Comput Biol. 2007 Oct 12;3(10):e193. doi: 10.1371/journal.pcbi.0030193 (PMC2014794; doi:10.1371/journal.pcbi.0030193)

A:

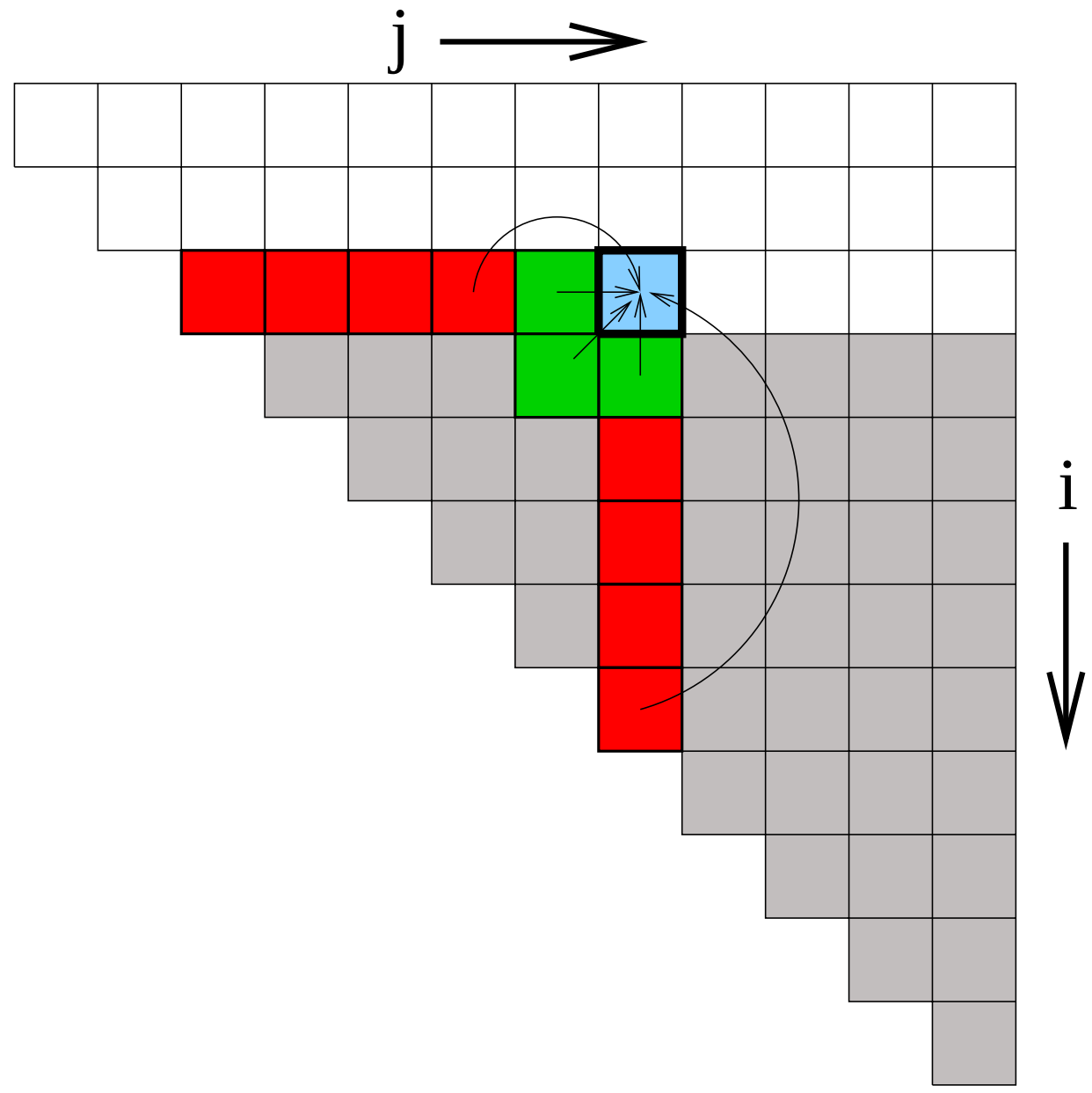

B:

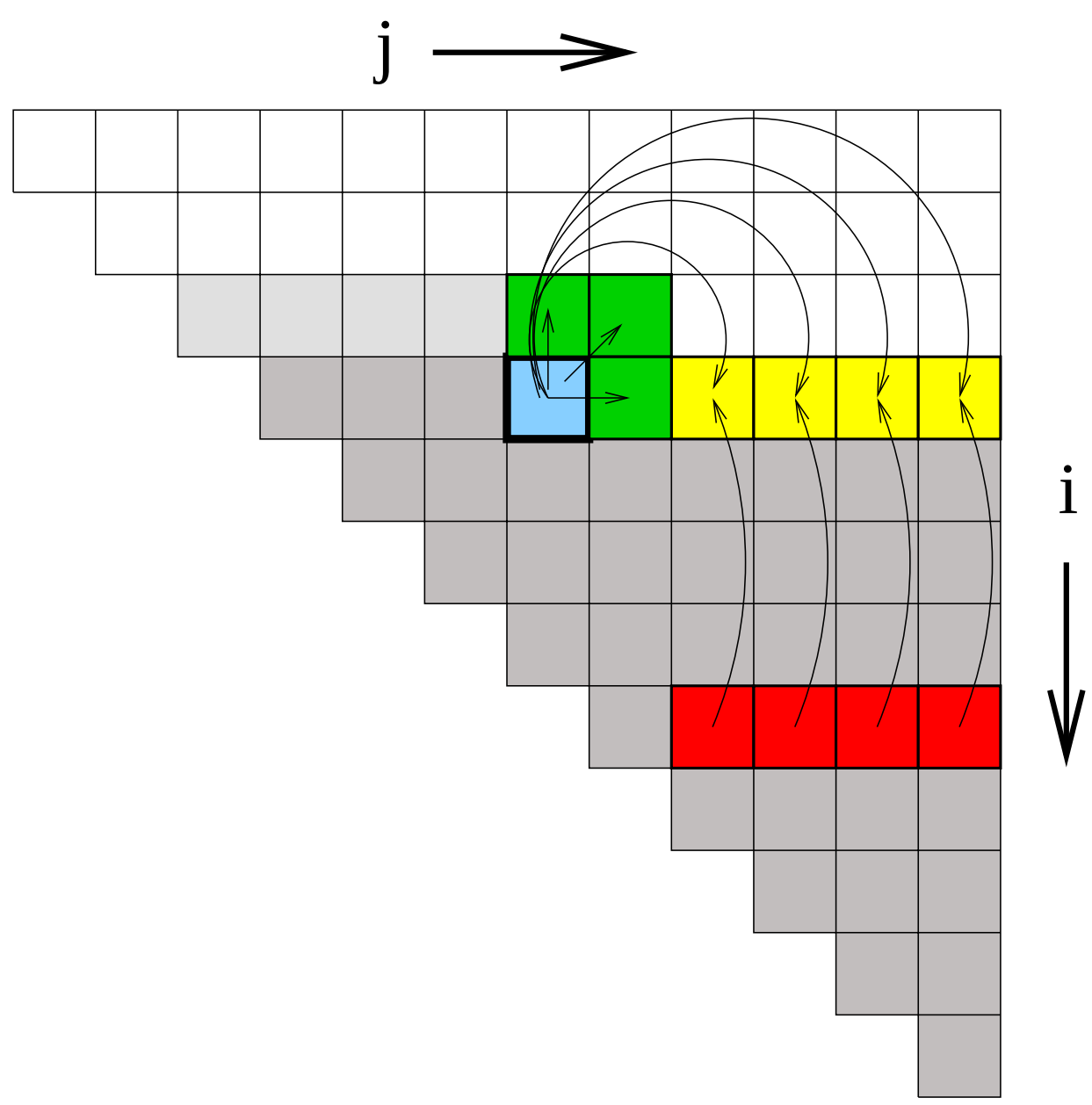

Supplement: Supplementary file 4 [file pcbi.0030193.sg003.pdf]
