# Supplementary material for: Fast Pairwise Structural RNA Alignments by Pruning of the Dynamical Programming Matrix
Source: PLoS Comput Biol. 2007 Oct 12;3(10):e193. doi: 10.1371/journal.pcbi.0030193 (PMC2014794; doi:10.1371/journal.pcbi.0030193)
Supplement: Supplementary file 5 [file pcbi.0030193.st001.pdf]

C_{mblend}	The cost of closing a multibranched loop
$C_{mblhelix}$	The cost of adding a stem to the multibranch loop (not including the first stem)
C_{mblnuc}	The cost of adding a single stranded nucleotide to a multibranched loop
C_{nGC}	The non-GC stem end cost
D	The score of an alignment. Not corrected for external single stranded nucleotides
D'	The alignment score. Corrected for external single stranded nucleotides
I sequence	One of the two sequences. Usually the longest
K sequence	The other sequence. Usually the shortest
L_{bl}	Bulge length cost
L_{il}	Internal loop length cost. Includes the asymmetry cost.
L_{hp}	Hairpin length cost
μ_1	Length of the single stranded region upstream of the last basepair in the I sequence
μ_2	Length of the single stranded region downstream of the last basepair in the I sequence
μ_3	Length of the single stranded region upstream of the last basepair in the K sequence
μ_4	Length of the single stranded region downstream of the last basepair in the K sequence
R_{bp}	Base pair substitution score
R_{ss}	Single strand substitution score
s	Stacking score
s_{hp}	Hairpin end stacking score
s_{il}	Internal loop end stacking score
σ	The state of an alignment

Table S1: Notation
